# Integration of single-cell sequencing and bulk RNA-seq to identify and develop a prognostic signature related to colorectal cancer stem cells

**DOI:** 10.1038/s41598-024-62913-3

**Published:** 2024-05-28

**Authors:** Jiale Wu, Wanyu Li, Junyu Su, Jiamin Zheng, Yanwen Liang, Jiansuo Lin, Bilian Xu, Yi Liu

**Affiliations:** 1 Guangdong Provincial Key Laboratory of Research and Development of Natural Drugs, School of Pharmacy, Guangdong Medical University, Zhanjiang, 524023 Guangdong China; 2 Well Lead Medical Co., Ltd., Guangzhou, 511434 Guangdong China; 3 School of Ocean and Tropical Medicine, Guangdong Medical University, Zhanjiang, 524023 Guangdong China; 4 Department of Biochemistry and Molecular Biology, School of Basic Medical Sciences, Guangdong Medical University, Dongguan, 523808 Guangdong China

**Keywords:** Colorectal cancer, Colorectal cancer stem cell, Single-cell transcriptome sequencing, Prognostic signature, RPS17, Cancer stem cells, Tumour biomarkers

## Abstract

The prognosis for patients with colorectal cancer (CRC) remains worse than expected due to metastasis, recurrence, and resistance to chemotherapy. Colorectal cancer stem cells (CRCSCs) play a vital role in tumor metastasis, recurrence, and chemotherapy resistance. However, there are currently no prognostic markers based on CRCSCs-related genes available for clinical use. In this study, single-cell transcriptome sequencing was employed to distinguish cancer stem cells (CSCs) in the CRC microenvironment and analyze their properties at the single-cell level. Subsequently, data from TCGA and GEO databases were utilized to develop a prognostic risk model for CRCSCs-related genes and validate its diagnostic performance. Additionally, functional enrichment, immune response, and chemotherapeutic drug sensitivity of the relevant genes in the risk model were investigated. Lastly, the key gene RPS17 in the risk model was identified as a potential prognostic marker and therapeutic target for further comprehensive studies. Our findings provide new insights into the prognostic treatment of CRC and offer novel perspectives for a systematic and comprehensive understanding of CRC development.

## Introduction

Colorectal cancer (CRC) is a prevalent and life-threatening malignancy on a global scale, characterized by considerable morbidity and mortality. Projections for 2023 anticipate approximately 153,020 new CRC cases and 52,550 CRC-related fatalities in the United States. Notably, the incidence rates in individuals below 65 years old have exhibited an annual increase of 2–3% since 2010, while metastatic CRC has shown a rise of 0.5–3% annually^1,2^. The growing population of CRC patients stems from diverse factors such as dietary habits, environmental conditions, pharmaceutical effects, and the emergence of early-onset diseases, alongside other underrecognized influencers^3^. The pronounced heterogeneity of CRC cells coupled with their heightened metastatic potential represents key characteristics of CRC, posing notable complexities in the clinical realms of diagnosis and prognosis^4,5^. Therefore, the development of a novel and effective diagnostic prognostic biomarker for CRC patients is urgently required^6,7^.

Cancer stem cells (CSCs), a small subset of cancer cells with strong self-renewal capacity, low differentiation and high tumorigenicity^8,9^. These cells are intricately linked to CRC’s heterogeneity^10^, metastasis^11^, and drug resistance^12^, facilitating tumor growth, spread, and adaptation to treatment^13,14^. Colorectal cancer stem cells (CRCSCs) play an important role in promoting CRC growth and progression^15^. In Kumar et al.’s study^16^, it was found that PIK3C3 improved the sensitivity of CRC treatment by inhibiting CRCSCs. Nie et al.’s study^17^ demonstrated that LRP5, through activation of the classical Wnt/β-catenin and IL-6/STAT3 signaling pathways, promoted CRCSCs, thereby increasing CRC tumorigenicity and drug resistance. Based on the importance of CRCSCs in the CRC tumor microenvironment (TME), the development of prognostic biomarkers associated with CRCSCs may become an effective tool to address the diagnostic and prognostic issues of CRC patients.

In the TME, CSCs intricately interact with diverse cell types such as immune cells (e.g., T cells, macrophages), stromal cells (e.g., fibroblasts), and endothelial cells. These interactions influence tumor biology via direct contact, paracrine signaling, and extracellular matrix (ECM) remodeling^18,19^. For example, CSC-induced macrophage polarization amplifies their distinctive attributes^20,21^. While myofibroblast-derived signaling molecules contribute to non-CSC dedifferentiation and chemoresistance in cancer cells^22^. Additionally, endothelial cells modulate the phenotype and chemoresistance of CRCSCs via NANOGP8 expression, regulated through the AKT pathway^23^. Such interactions highlight the vital role of CSCs within the tumor's cellular network, exerting a significant impact on cancer progression and treatment responses.

Single-cell sequencing (scRNA-seq) technology has emerged as a revolutionary genomic tool capable of revealing gene expression and genomic information at the level of individual cells^24^. This technology provides reliable research methods for clinical cancer treatment studies^25,26^ and offers insights into tumor heterogeneity and genomic variations, elucidating principles behind cancer relapse and metastasis^27,28^. Compared to traditional transcriptomics, scRNA-seq will provide deeper cellular-level transcriptome analysis of CRC^29,30^. Additionally, it facilitates investigation into the roles and impacts of CRCSCs within TME, paving the way for the discovery of novel biomarkers.

**Figure 1:**
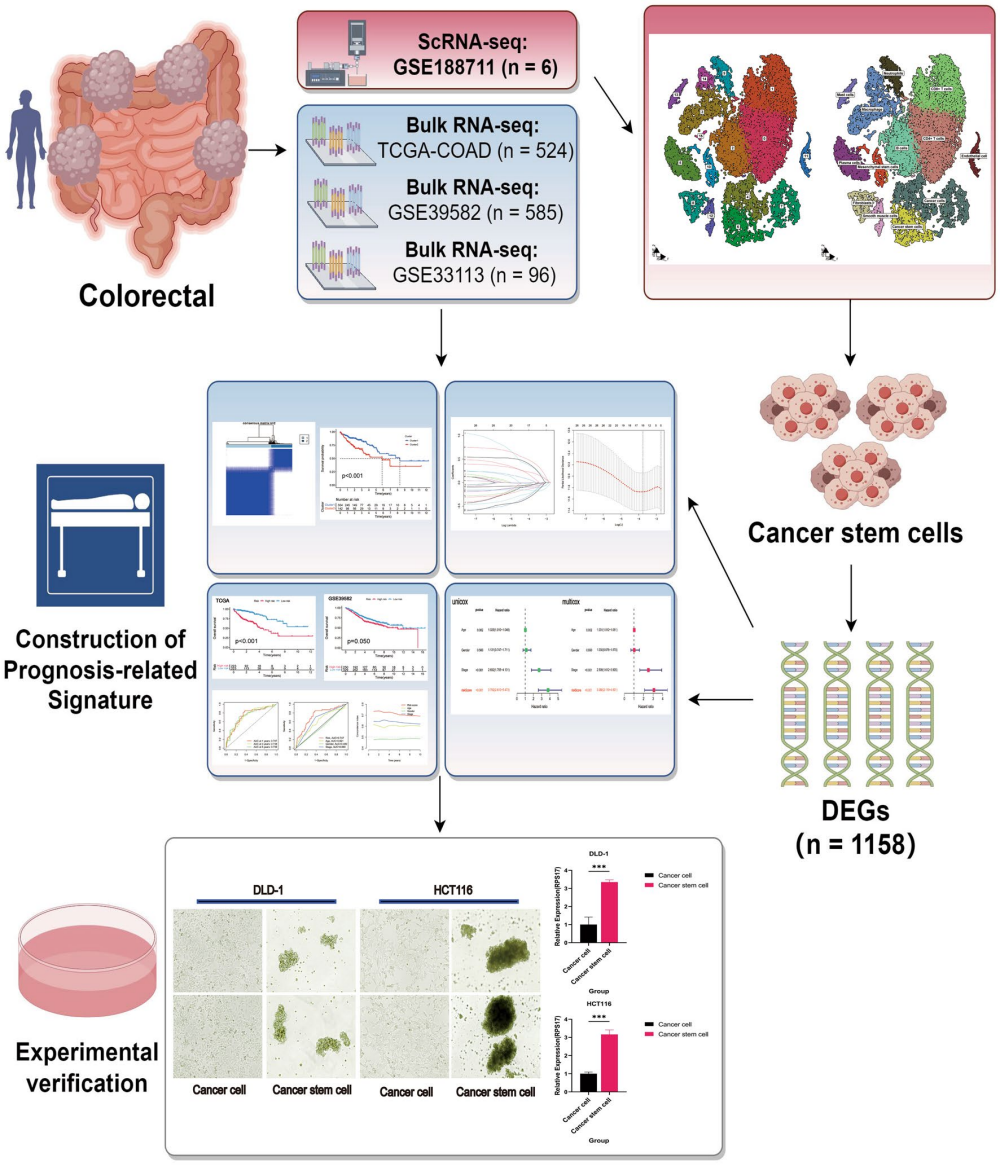
Flowchart material for this study was drawn by Figdraw.

Utilizing single-cell transcriptome sequencing integrated with bulk transcriptome and clinical data, this study mapped the CRCSCs landscape in the CRC microenvironment (Fig. 1). We constructed and validated a prognostic risk model based on CRCSC-associated genes, identifying clinically relevant genes that may refine CRC patient outcomes and elucidate disease mechanisms.

## Results

### Identification of CRCSCs and gene distribution

**Figure 2:**
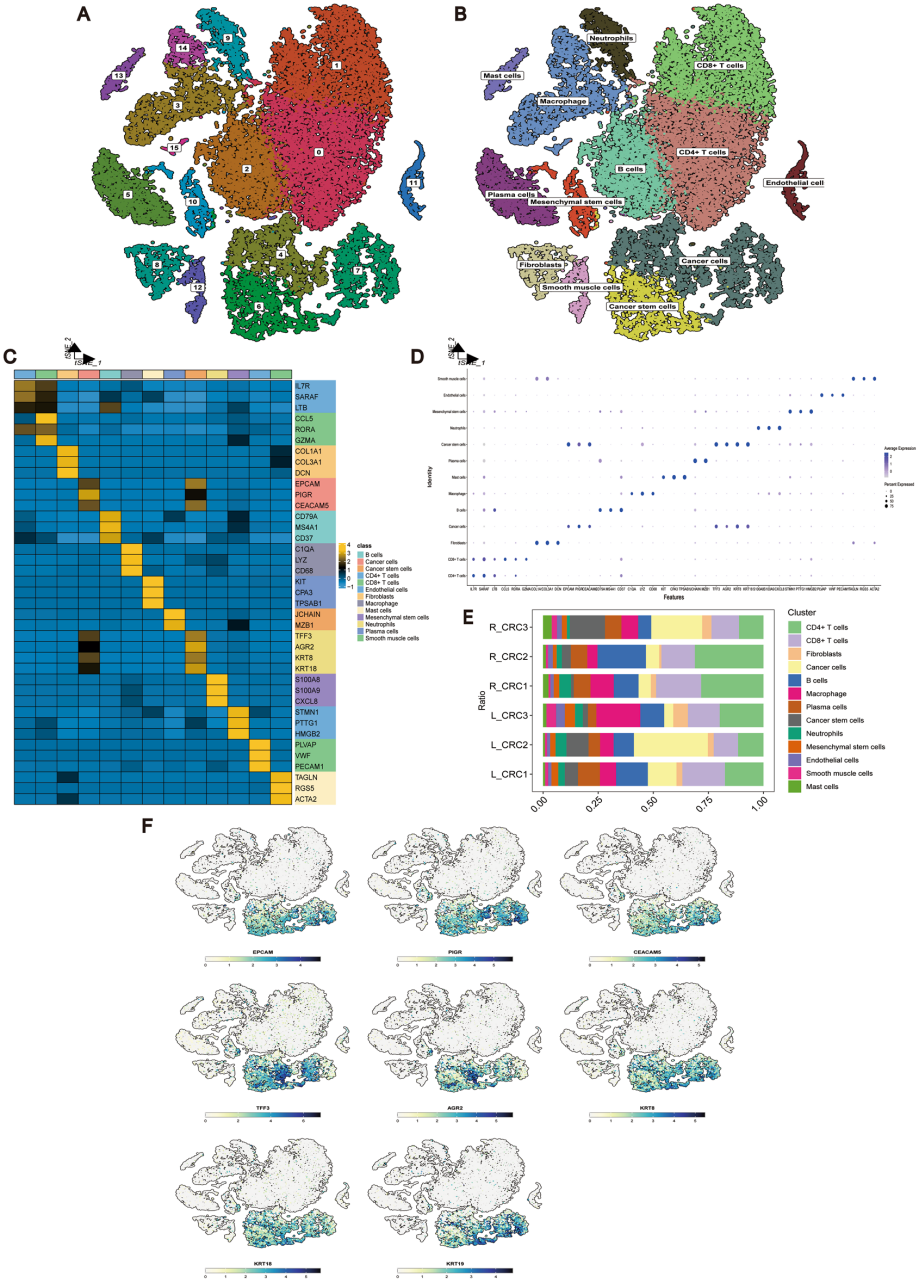
scRNA-seq to identify cell types of CRC samples. (**A**) scRNA-seq data yielded t-SNE plot for 15 Clusters. (**B**) scRNA-seq data yielded t-SNE plot for 13 cell types. (**C**) Heatmap showing markers for 13 cell types. (**D**) Scatterplot showing markers for 13 cell types. (**E**) Distribution of 13 cell types in different samples of scRNA-seq data. (**F**) Cancer cells and CSCs marker gene t-SNE plot. 13 cell types of distribution plot.

We first used GSE188711 to distinguish cell subpopulations, including CRCSCs in the CRC-TME. After a series of filtering the data for original technological noise, we clustered the cells in the sample into 15 cell subpopulations (Fig. 2A). Based on the marker genes expressed by different cells (CSCs (TFF3^31^, AGR2^32^, KRT8^33^, KRT18^34,35^), cancer cells (EPCAM^36^, PIGR^37^, CEACAM5^38^), CD4^+^ T cells (IL7R^39^, SARAF^40^, LTB^41^), CD8^+^ T cells (CCL5^42^, RORA^43^, GZMA^44^), fibroblasts (COL1A1^45^, COL3A1^45^, DCN^46^), B cells (CD79A^47^, MS4A1^48^, CD37^49^), macrophages (C1QA^50^, LYZ^51^, CD68^52^), mast cells (KIT^53^, CPA3^54^, TPSAB1^55^), plasma cells (JCHAIN^42^, MZB1^42^), neutrophils (S100A8^56^, S100A9^57^, CXCL8^58^), mesenchymal stem cells (STMN1^59^, PTTG1^60^, HMGB2^61^), endothelial cells (PLVAP^62^, VWF^63^, PECAM1^64^), smooth muscle cells (TAGLN^65^, RGS5^66^, ACTA2^67^), we identified 13 cell types in the CRC samples (Fig. 2B), with the expression of their marker genes as shown in Fig. 2C,D. The proportions of the different cell types in the samples were illustrated in Fig. 2E. Additionally, Fig. 2F demonstrates the variation in the distribution of marker genes between CRCSCs and cancer cells in CRC tissues.

### Cell communication analysis

**Figure 3:**
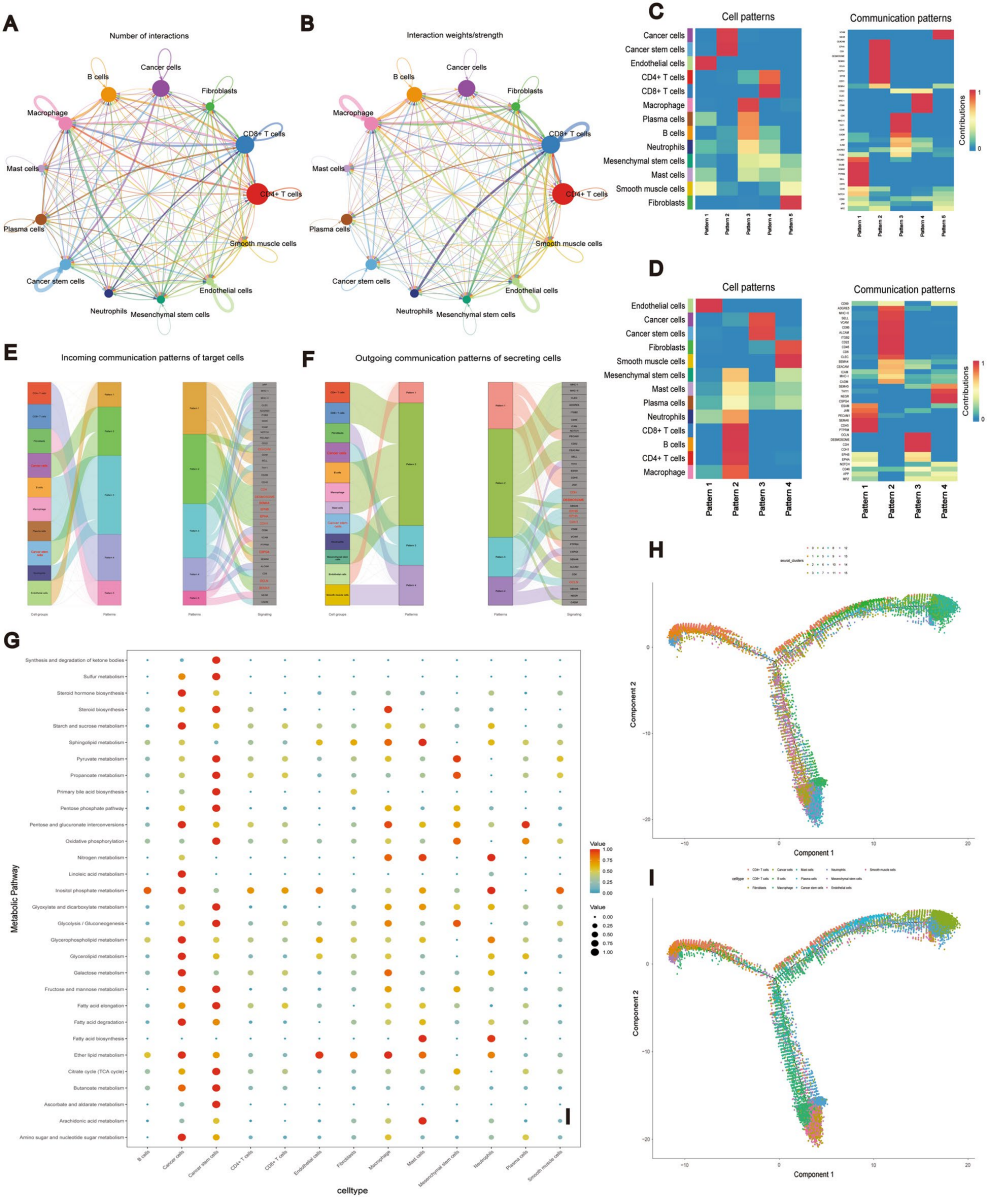
Analysis of cellular communication, metabolism, and differentiation. (**A**) Circle diagram of the number of interactions of the 13 cell types. (**B**) Circle diagram of the specific gravity of interactions of the 13 cell types. (**C**) Heat map of the incoming pattern of signaling between the 13 cell types. (**D**) Heat map of the outgoing pattern of signaling between the 13 cell types. (**E**) Flow diagram of the incoming pattern of signaling between the 13 cell types; (**F**) conduction efferent pattern river diagram. (**G**) We scored the enrichment of KEGG metabolic pathways for 13 cell types and selected the top 30 metabolically relevant pathways for scatter plot presentation. (**H**) Mock time series analysis to explore the differentiation changes of 15 Clusters. (**I**) Mock time series analysis to explore the differentiation changes of 13 cell types, with Cancer stem cells as the starting point.

The intricate TME hosts numerous cellular interactions, and variations in inter-cellular roles may diminish the efficacy of tumor treatment^68,69^. Therefore, it is vital to investigate inter-cellular functions and mechanisms as a crucial prerequisite to clinical tumor treatment^70^. Our initial step involved examining the intercellular communication between CRCSCs and 13 specific cell types (Fig. 3A,B). Following an analysis of the quantity and weighted significance of these interactions, it became evident that CRCSCs primarily focused on correlating their biological functions with cancer cells and immune cells, specifically macrophages, B cells, and CD8^+^ T cells. Subsequently, we investigated the coordinated function of CRCSCs with multiple cell populations and pathways. This involved clustering based on two metrics, Cophenetic and Silhouette, resulting in the selection of five patterns for the afferent model and four for the efferent model (Supplementary Fig. 1). The afferent (incoming) model revealed the coordination between CRCSCs and cancer cells, as they clustered in pattern 2 and coordinated through the CEACAM, CDH, DESMOSOME, SEMA4, EPHA, EPHB, CDH1, CSPG4, OCLN, and SEMA5 signaling pathways in response to incoming signals (Fig. 3C). The efferent pattern results showed that CRCSCs and cancer cells coordinated with each other, clustered in pattern 3, which coordinated to drive communication by coordinating with the CDH, DESMOSOME, EPHA, EPHB, CDH1, and OCLN signaling pathways (Fig. 3D). Figure 3E,F display visualizations of the afferent and efferent patterns of signaling communication using river diagrams.

### Cellular metabolic function analysis and cell trajectory prediction

TME encompasses various cell types engaged in diverse metabolic processes, exerting a significant influence on tumor growth and treatment^71,72^. We computed the metabolic enrichment scores of the sorted samples and identified the top 30 active pathways, which are illustrated in scatter plots (Fig. 3G). Our findings revealed that CRCSCs exhibited high enrichment scores in 15 active pathways, including oxidative phosphorylation, glycolysis, fatty acid degradation and TCA cycle. The time-series analysis predicted the cell differentiation of 15 clusters with 13 cell types. The results indicated that, starting from CRCSCs, the main differentiation trajectories led towards cancer cells and fibroblasts (Fig. 3H,I), the results suggest that CSCs are more inclined to develop properties like those of cancer cells and fibroblasts during differentiation.

### Construction of a prognostic risk model for CRCSCs-related genes

**Figure 4:**
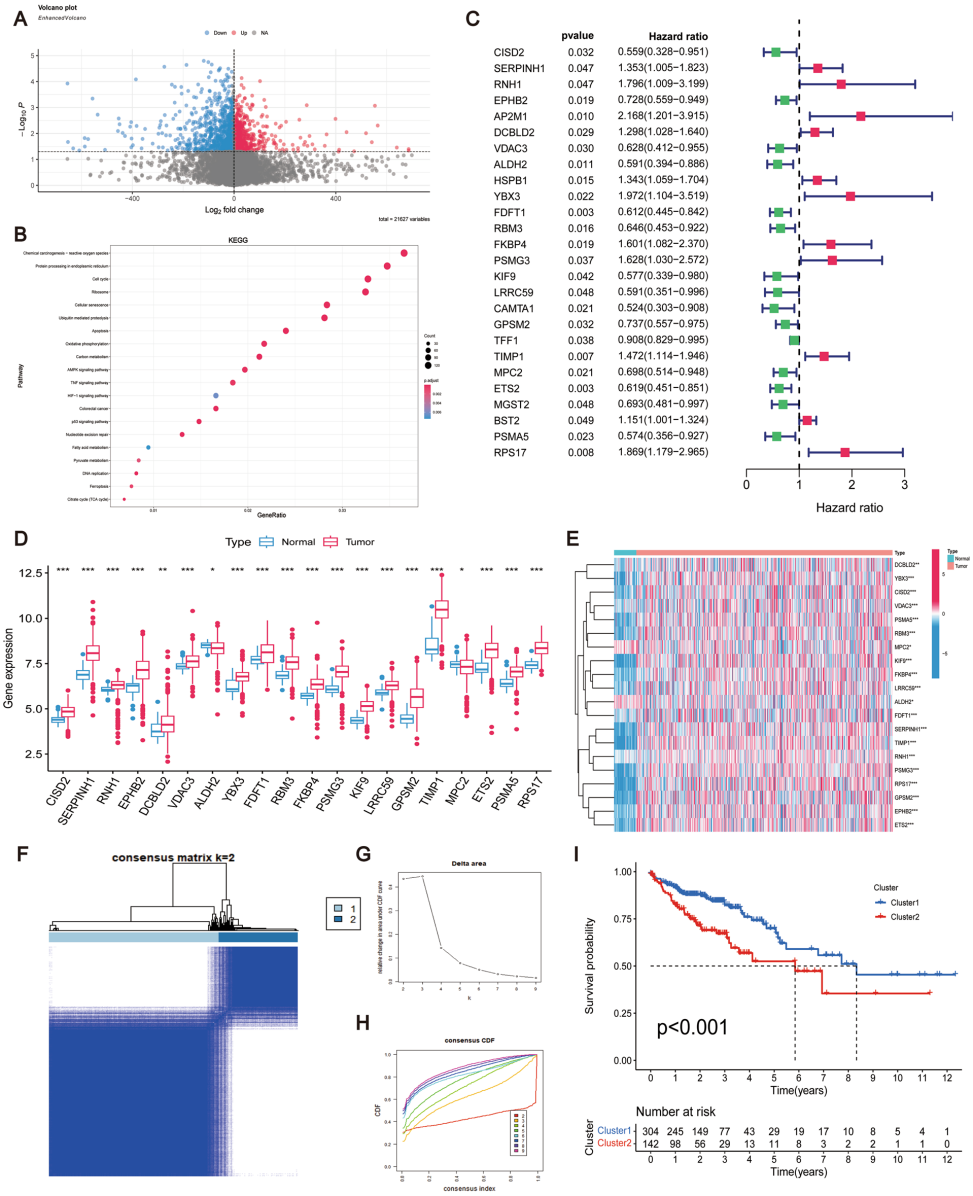
CRCSCs differential gene screening and survival significance study. (**A**) 1158 colorectal CSCs differential genes from scRNA-seq data for volcano plot visualization. (**B**) Scatterplot of GO functional analysis of differential genes. (**C**) Forest plot demonstrating the screening of 26 prognostically relevant genes after univariate COX regression scores (P < 0.05). Among the 26 genes associated with prognosis, 20 genes that were differentially expressed in CRC were extracted and shown as (**D**) box plots and (**E**) heat maps, respectively. (P < 0.05). (**F**) Prognosis-related consensus clustering matrix at K = 2. (**G**) Relative changes in the area under the CDF curves at K = 2–9. (**H**) Empirical CDF plots at K = 2–9. (**I**) Survival difference analysis between Cluster1 and Cluster2 (P < 0.05).

To investigate whether CRCSCs-related genes can serve as prognostic biomarkers for CRC, we initially identified 1158 differentially expressed genes associated with CRCSCs from the CSC subpopulation of a scRNA-seq dataset using the criteria of |logFC| = 0.5 and P < 0.05. Subsequently, we generated a volcano map based on the GSE33113 dataset (Fig. 4A) and performed further enrichment analysis of the differentially expressed genes using the KEGG pathway (R = 1, P < 0.05) (Fig. 4B). Next, we extracted differential gene expression data from the TCGA-COAD expression matrix and integrated it with clinical samples for conducting univariate Cox regression analysis using a significance threshold of P < 0.05, resulting in the identification of 26 genes associated with CRC prognosis (Fig. 4C).

To ensure the consistency of the data, we applied the “limma” package to screen the scRNA-seq and bulk RNA-seq data for common significantly different genes, and then selected 20 genes for in-depth analysis (Fig. 4D,E). Subsequently, we classified the 20 CRCSCs-related genes, which were previously identified to have prognostic diagnostic capability, into Cluster 1 and Cluster 2 based on the minimum overlapping expression levels of the genes at K = 2 and the lowest cumulative distribution function (CDF) values (Fig. 4F–H). The survival analysis results revealed significant differences between the subgroups of CRCSCs-related genes (Fig. 4I).

**Figure 5:**
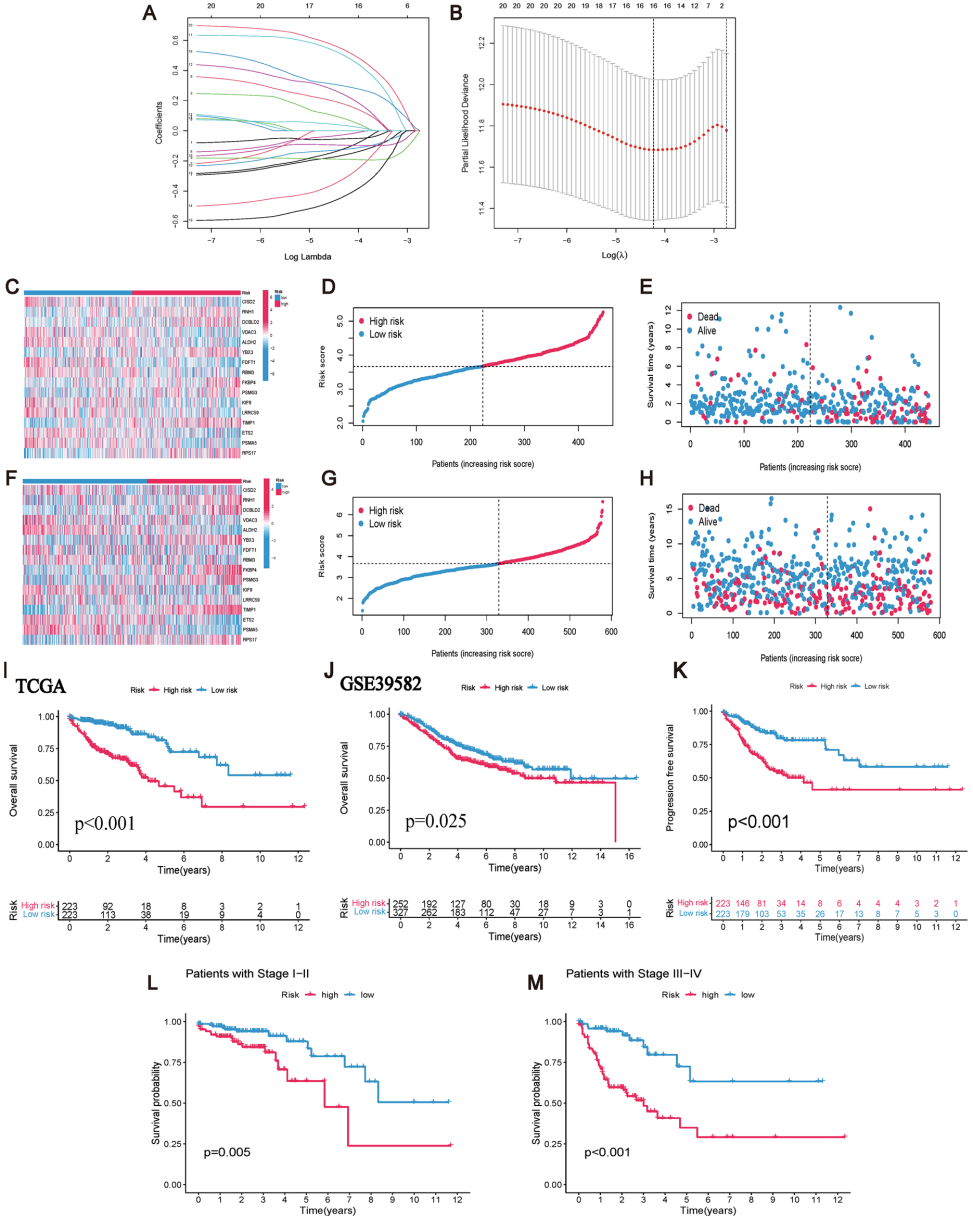
Construction of CRCSCs-related prognostic risk model. (**A**) Lasso regression screening of CRCSCs-related genes at the nadir of cross-validation. (**B**) Lasso regression trajectory of each independent variable. (**C**,**D**) Prognostic risk model scores differentiate the analysis of survival differences between high-risk and low-risk groups, with TCGA as the Training group and GSE39582 as the Testing group, the overall survival of patients in the high-risk group was significantly lower than that in the low-risk group (P < 0.05). (**E**) The progression-free survival analysis of the prognostic risk model was also significantly different. (**F**,**G**) and (**H**) show the risk heatmap, the risk score plot, and the scatterplot of the risk distribution for the Training group, respectively. While (**I**), (**J**), and (**K**) show the risk heatmap, risk score curve plot, and risk distribution scatter plot for the Testing group. (**L**) Shows the survival difference analysis between the high-risk group and the low-risk group within the clinical stage I–II (P < 0.05). (**M**) Shows the survival difference analysis between the high-risk group and the low-risk group within the clinical stage III-IV (P < 0.05).

We utilized Lasso-Cox regression analysis to select 16 genes (CISD2, RNH1, DCBLD2, VDAC3, ALDH2, YBX3, FDFT1, RBM3, FKBP4, PSMG3, LRRC59, KIF9, TIMP1, ETS2, PSMA5, and RPS17) for prognostic CRCSCs-related constructing the risk model (P < 0.05) (Fig. 5A,B). Comparative clinical statistical analyses conducted on the Train and Test groups are presented in Table 1. Notably, a substantial disparity in CRC T-stage compared to M-stage exists between these cohorts. This observed distinction likely mirrors variations in disease severity and prognostic outcomes among the groups, thereby indicating a potential necessity for enhancing the model's generalizability across diverse patient populations. The correlations among the gene scores of the risk model are shown in Table 2.

**Table 1:** Clinical statistics analysis for prognostic risk modeling (train and test).

Covariates	Type	Total	Test	Train	Pvalue
Age	< = 65	402 (40.52%)	219 (40.11%)	183 (41.03%)	0.795
	> 65	590 (59.48%)	327 (59.89%)	263 (58.97%)	
Gender	Female	462 (46.57%)	250 (45.79%)	212 (47.53%)	0.6089
	Male	530 (53.43%)	296 (54.21%)	234 (52.47%)	
Stage	Stage I–II	544 (54.84%)	294 (53.85%)	250 (56.05%)	0.2719
	Stage III-IV	437 (44.05%)	252 (46.15%)	185 (41.48%)	
	Unknown	11 (1.11%)	0 (0%)	11 (2.47%)	
T	T1–2	146 (14.72%)	60 (10.99%)	86 (19.28%)	3.00E−04
	T3–4	845 (85.18%)	486 (89.01%)	359 (80.49%)	
	Unknown	1 (0.1%)	0 (0%)	1 (0.22%)	
M	M0	815 (82.16%)	486 (89.01%)	329 (73.77%)	0.0384
	M1	121 (12.2%)	60 (10.99%)	61 (13.68%)	
	Unknown	56 (5.65%)	0 (0%)	56 (12.56%)	
N	N0	570 (57.46%)	305 (55.86%)	265 (59.42%)	0.1096
	N1	238 (23.99%)	136 (24.91%)	102 (22.87%)	
	N2	178 (17.94%)	99 (18.13%)	79 (17.71%)	
	N3	6 (0.6%)	6 (1.1%)	0 (0%)

**Table 2:** Correlation coefficients of 16 genes constituting the prognostic risk model associated with CRCSCs after Lasso–Cox regression.

Gene	Coef
CISD2	− 0.0525091127452745
RNH1	0.0748604109350036
DCBLD2	0.0335933671405521
VDAC3	− 0.0228121627253004
ALDH2	− 0.15644654512311
YBX3	0.170463350665453
FDFT1	− 0.189104443036247
RBM3	− 0.121052458851655
FKBP4	0.488974077652429
PSMG3	0.229497946411793
KIF9	− 0.11139442718464
LRRC59	− 0.304301568182604
TIMP1	0.293337834574802
ETS2	− 0.0956355325088187
PSMA5	− 0.424578983620908
RPS17	0.527004831049996

Risk scores were derived from the expression profiles and correlations of 16 CRCSCs genes, allowing classification of TCGA patients (training group) into high- and low-risk strata at the median. Figure 5C–E depicts the heatmap, risk curve, and scatter plot for risk distribution within the training group. Post-data consolidation, GSE39582 served as the validation group, applying the same risk criteria from TCGA for group stratification. The heatmap, risk curve, and scatter plot for the validation group’s risk distribution are presented in Fig. 5F–H. Survival analyses confirmed a pronounced disparity in survival rates, with high-risk patients exhibiting significantly reduced survival compared to their low-risk counterparts (TCGA: P < 0.001; GSE39582: P = 0.025), as illustrated in Fig. 5I,J. The prognostic model, based on TCGA data, demonstrated a highly significant difference in progression-free survival (P < 0.001), as shown in Fig. 5K. Subsequent survival analyses with different clinical stages indicated that patients in the low-risk group survived significantly longer than those in the high-risk group (stage I–II: P = 0.005, stage III–IV: P < 0.001) (Fig. 5L,M). These results suggest that our prognostic risk model constructed using genes related to CRCSCs may have favorable results for the prognosis of CRC patients.

### Validation of prognostic risk model for CRCSCs-related genes

**Figure 6:**
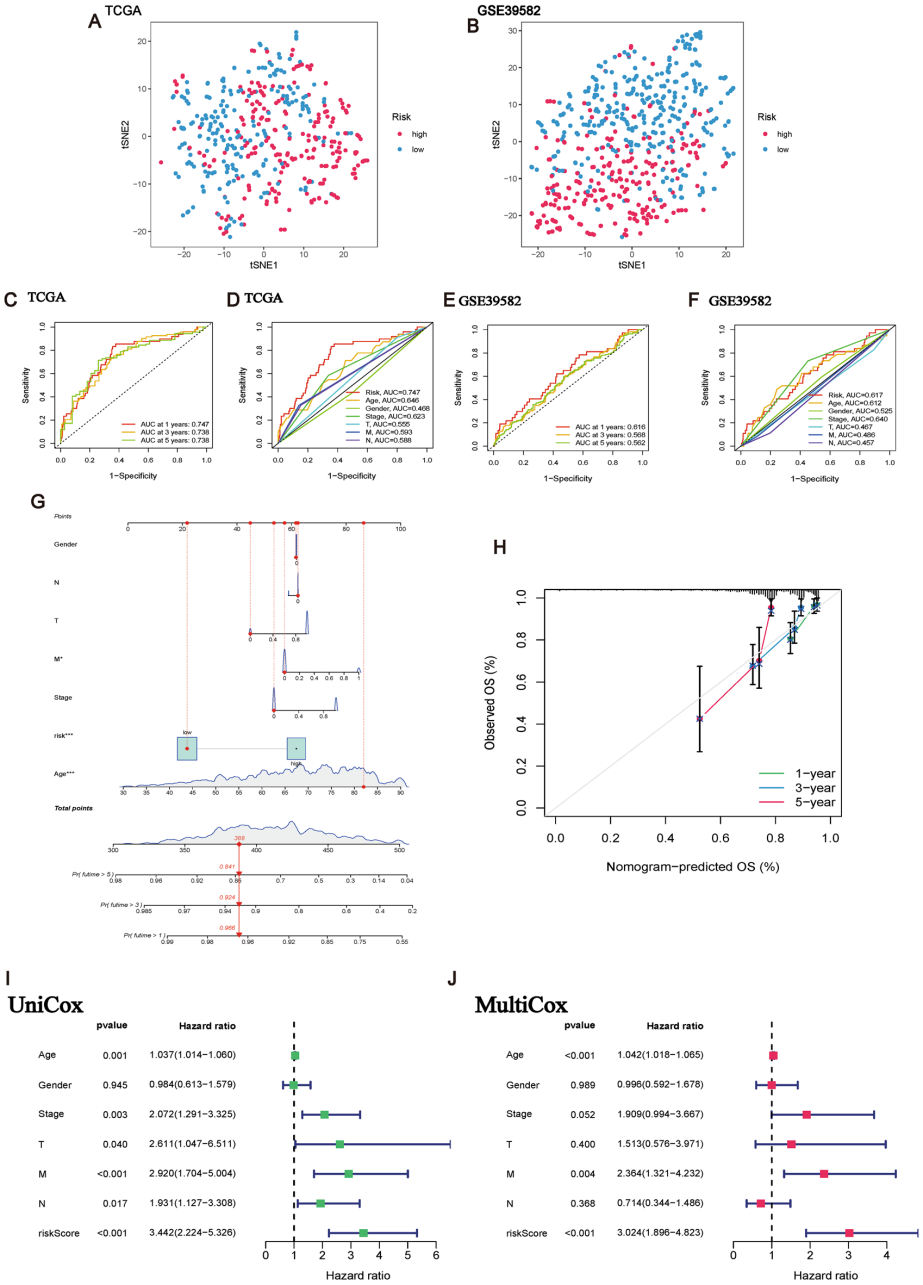
Validation of the prognostic performance of the prognostic risk model. (**A**) Principal component analysis demonstrating the ability of the risk model scores to discriminate between samples in the Training group. (**B**) Principal component analysis demonstrating the ability of the risk model scores to discriminate between samples in the Testing group. (**C**) The TCGA dataset yields ROC curves validating the predictive performance of the risk model over the 1-year, 3-year, and 5-year periods, with AUCs of 0.747, 0.738, and 0.738, respectively. (**D**) ROC curves incorporating traditional clinical factors in the TCGA group validated the predictive performance of the risk model, with risk scores: 0.747; age: 0.646; gender: 0.468; stage: 0.623; T: 0.555; M: 0.593; N: 0.588. (**E**) The GEO dataset yields ROC curves validating the predictive performance of the risk model over the 1-year, 3-year, and 5-year periods, with AUCs of 0.616, 0.568 and 0.562, respectively, respectively. (**F**) ROC curves incorporating traditional clinical factors in the GEO group validated the predictive performance of the risk model, with Risk Score: 0.617; Age: 0.612; Gender: 0.525; Stage: 0.640; T: 0.467; M: 0.486; N: 0.457. (**G**) Nomogram plot to validate that the risk model scores with good prognostic performance. (**H**) Standard curve showing that 1-, 3-year performance would be more accurate than 5-year. (**I**) Successively combined traditional clinical factors in univariate Cox regression (HR = 3.442 (2.224–5.376)) and (**J**) multifactorial Cox regression analyses (HR = 3.024 (2.119–4.823)), P < 0.001.

Firstly, the results of our principal component analysis were used to validate the clustering results of the high-risk group versus the low-risk group (Fig. 6A,B), suggesting that the median risk score can effectively stratify patients. The results showed that the areas under the ROC curves associated with the prognostic risk model for CRCSCs were 0.747, 0.738, and 0.738 at 1, 3, and 5 years, respectively, implying that they all had good prognostic performance over the 5-year period (Fig. 6C). In addition, we analyzed the risk score in combination with conventional clinical factors of CRC patients, and the results showed that the prognostic diagnostic accuracy of the risk model surpasses that of other clinical factors (risk score: 0.747; age: 0.646; gender: 0.468; stage: 0.623; T: 0.555; M: 0.593; N: 0.588) as depicted in Fig. 6D. In Fig. 6E,F, the ROC curves illustrate higher values for the risk model at 1 year (0.616), 3 years (0.568), and 5 years (0.562) compared to the respective clinically relevant ROC curves (Risk Score: 0.617; Age: 0.612; Sex: 0.525; Stage: 0.640; T: 0.467; M: 0.486; N: 0.457) based on the GEO dataset.

As shown in Fig. 6G,H, the prediction accuracy was higher within 1 (0.966), 3 (0.924) years compared to 5 years (0.841), and the standardized curves validated the predictive results of the Nomogram. We then performed univariate Cox regression analysis (risk score: HR = 3.442 (2.442–5.326)) (P < 0.001) (Fig. 6I) and multivariate Cox regression analysis (risk score: HR = 3.024 (1.896–4.823)) (P < 0.001)) (Fig. 6J) for risk score in combination with other clinical factors in sequence. The risk ratios were higher than Age, M stage, etc., and the results suggest that our prognostic risk model based on the correlation of CRCSCs has the potential to be an independent and effective prognostic biomarker.

### Functional enrichment of CRCSCs-related genes

**Figure 7:**
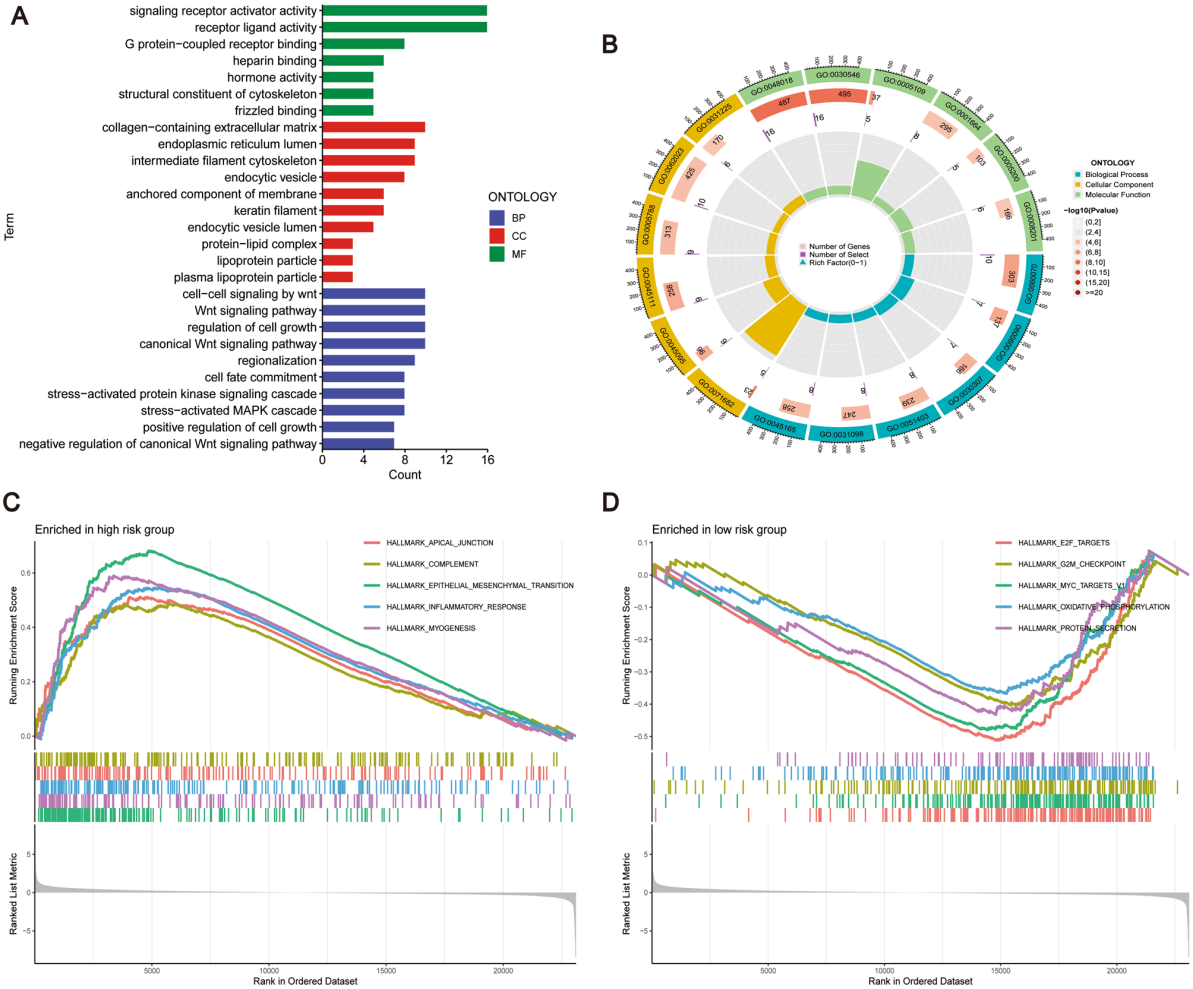
Functional analysis of the prognostic risk model. (**A**) Results of GO functional enrichment analysis showed (P < 0.05, R = 1) that the biological processes of the risk model are active in the Wnt pathway, the cellular fractions are enriched in collagen-containing extracellular matrix, and the molecular functions are active in signaling receptor activator activities. (**B**) Showing the corresponding circle diagrams. (**C**) and (**D**) show the Hallmark enrichment analysis of the patients of high-risk group and the patients of low-risk group, respectively. As a result, high-risk patients were mainly enriched for EMT process, and low-risk patients were negatively correlated with E2F activity.

To investigate the primary biological functions of CRCSCs-related gene risk models, we conducted GO functional and Hallmark analyses comparing high-risk patients with low-risk patients. The GO enrichment analyses (Fig. 7A,B) revealed that risk models are mainly enriched for the Wnt pathway (including cell–cell signaling by Wnt, canonical Wnt signaling pathway) and regulation of cell growth during BP, for collagen-containing extracellular matrix in CC, and Signaling receptor activator activity and receptor ligand activity in MF.

The results of the Hallmark analysis of CRC patients are shown in Fig. 7C,D. High-risk group patients exhibited significant enrichment in Apical junction, Complement, Epithelial mesenchymal transition (EMT), Inflammatory response and Myogenesis, with the highest enrichment score observed for EMT (highest enrichment score of 0.6). This suggests that high-risk patients identified by the risk model are more likely to undergo EMT, indicating a potentially more severe CRC disease^73,74^. In contrast, low-risk patients showed enrichment for E2F targets, G2M checkpoint, Myc targets V1, Oxidative phosphorylation, and Protein secretion, with E2F TARGETS exhibiting the lowest enrichment score (highest enrichment score of − 0.5). It was shown that reduced E2F expression decreases the growth and invasive capacity of cancer cells^75,76^, implying a potentially more stable TME in low-risk patients identified by our constructed risk score.

### Immunoassay of CRCSCs-related genes

**Figure 8:**
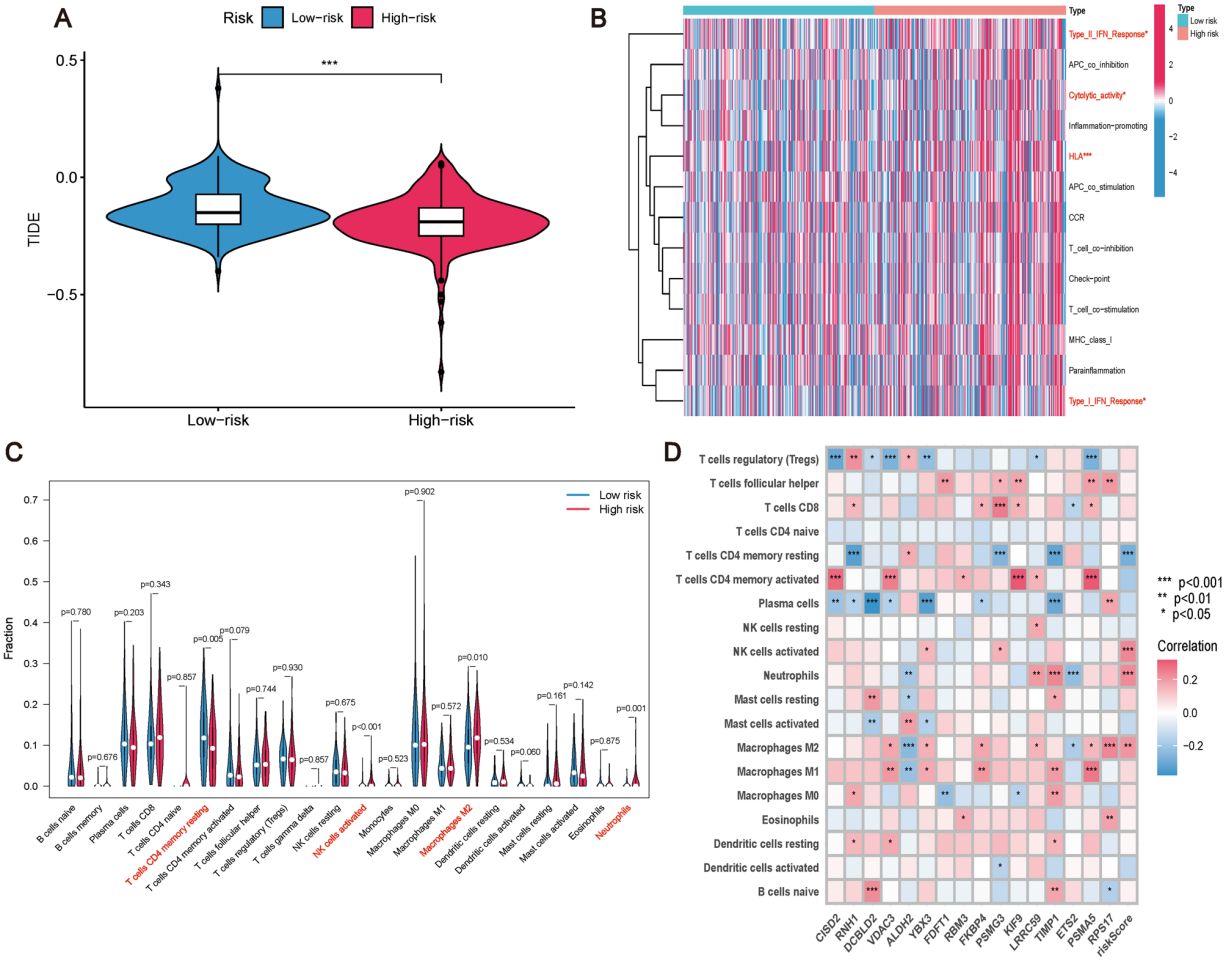
Immunological correlation analysis of prognostic risk models. (**A**) TIDE scores between high-risk and low-risk groups, TIDE scores of patients in high-risk group were significantly lower than those of patients in low-risk group. (**B**) Immunological correlation responses predicting prognostic risk models were enriched for the presence of T cells CD4 memory resting, NK cells activated, Cytolytic activity, and HLA. (**C**) Immune infiltration of the prognostic risk model with significant differences in T cells CD4 memory resting, NK cells activated, Macrophages M2, and Neutrophils, and (**D**) Immunological infiltration of the 16 CRCSCs-related genes comprising the prognostic risk model on immune infiltration correlation.

Given that the cell communication analysis and Hallmark results demonstrate a strong correlation between the risk model and immune function, we further investigate immune-related prognostications. The TIDE scores of high-risk patients were significantly lower than those of low-risk patients in our risk model predictions (Fig. 8A), suggesting that high-risk patients have a lower capacity to undergo immune evasion and rejection, and that the efficacy of using immunotherapy in high-risk patients may be better. Furthermore, the ssGSEA analysis of immune-related reactions showed that patients in the high-risk group were enriched in type I IFN response, type II IFN response, cell lytic activity, and HLA response (Fig. 8B).

Analysis of immune infiltration reveals that high-risk patients exhibit a decreased proportion of “T cell CD4 memory resting cells” compared to their low-risk counterparts, implying a potentially compromised tumor-fighting capacity within the immune system of high-risk individuals. Moreover, the frequencies of “activated NK cells,” “M2 macrophages,” and “neutrophils” are markedly elevated in the high-risk group relative to the low-risk group, indicating the presence of an inflammatory milieu and immune-suppressive traits within the TME (P < 0.05) (Fig. 8C). Additionally, we established the correlation between CRCSCs-related genes and immune cells (Fig. 8D). These findings suggest that our risk model can effectively evaluate the impact of immunotherapy in patients.

### Drug sensitivity analysis

**Figure 9:**
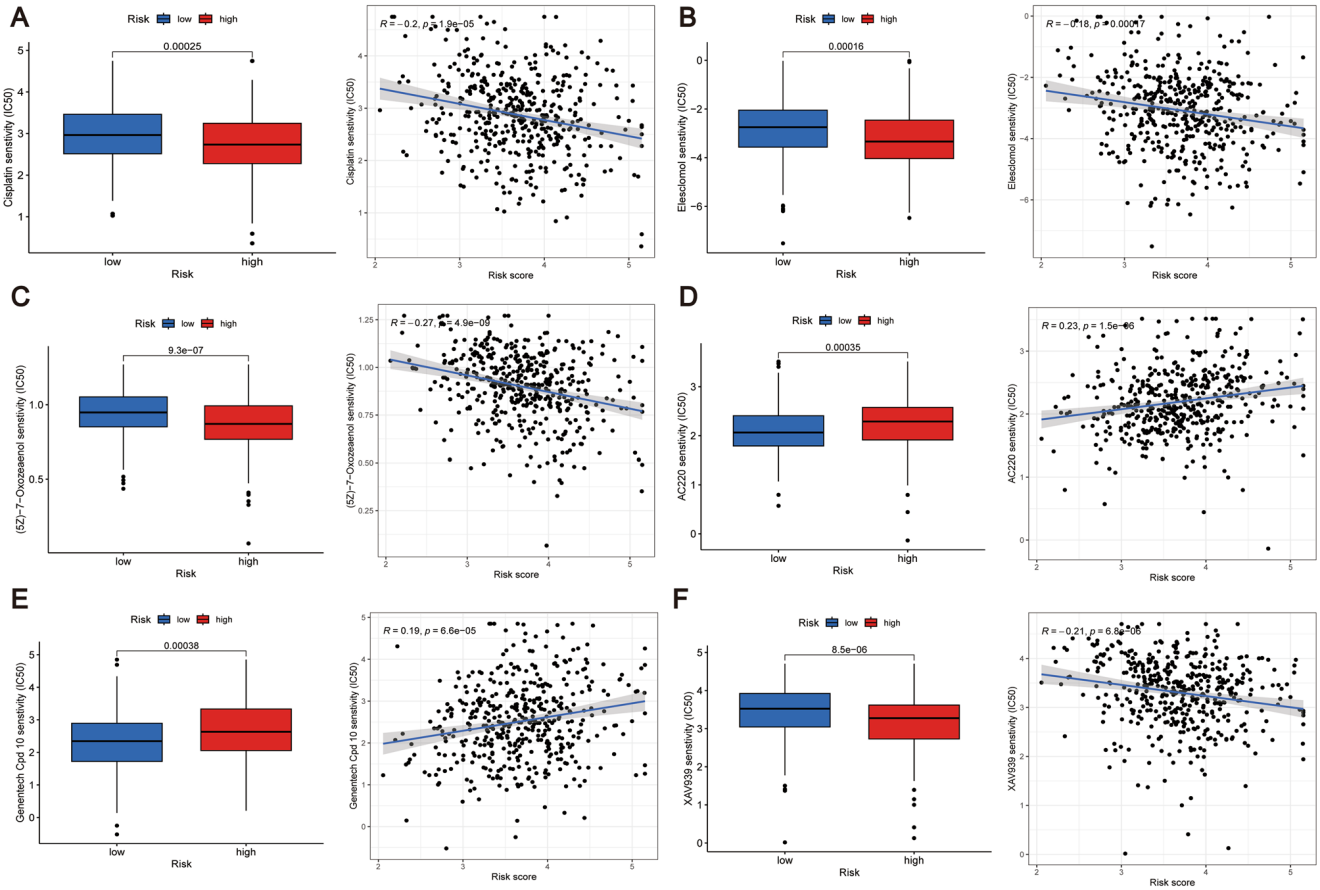
Chemotherapeutic drug sensitivity analysis. (**A**–**F**) There was a significant difference in the sensitivity of the high-risk and low-risk groups, as differentiated by the prognostic risk model scores, to cisplatin, Esketamine, (5Z)-7-Oxozeaenol, AC220 (Quizartinib), Genentech Cpd 10, and XAV939 with cisplatin, (5Z)-7-Oxozeaenol, Esketamine, and XAV939 were more sensitive in patients in the high-risk group.

To investigate the potential of risk models in assessing the resistance of CRC patients to clinical chemotherapeutic agents, we obtained 14 chemotherapeutic agents associated with the risk model using the “pRRophetic” R software package prediction (Supplementary Fig. 2). Figure 9A–F show some of the results, showing increased sensitivity to chemotherapeutic agents such as cisplatin in high-risk patients, i.e., indicating that high-risk patients differentiated by our constructed prognostic model had higher drug sensitivity to Cisplatin (*R* = − 0.2, P < 0.001), Elesclomol (*R* = − 0.18, P < 0.001), (5Z)-7-oxozeaenol (*R* = − 0.27, P < 0.001), and XAV939 (*R* = − 0.21, P < 0.001), while to AC220 (*R* = 0.23, P < 0.001) and Genentech Cpd 10 (*R* = 0.19, P < 0.001) the drug sensitivity was lower than that of the low-risk group, and these results may provide some reference value for patients in the high- and low-risk groups in the selection of clinical chemotherapy regimens may provide some reference value, such as the selection of chemotherapeutic agents, the measurement of the dose of chemotherapeutic agents used, and other aspects.

### Key gene screening, validation, and clinical relevance analysis

**Figure 10:**
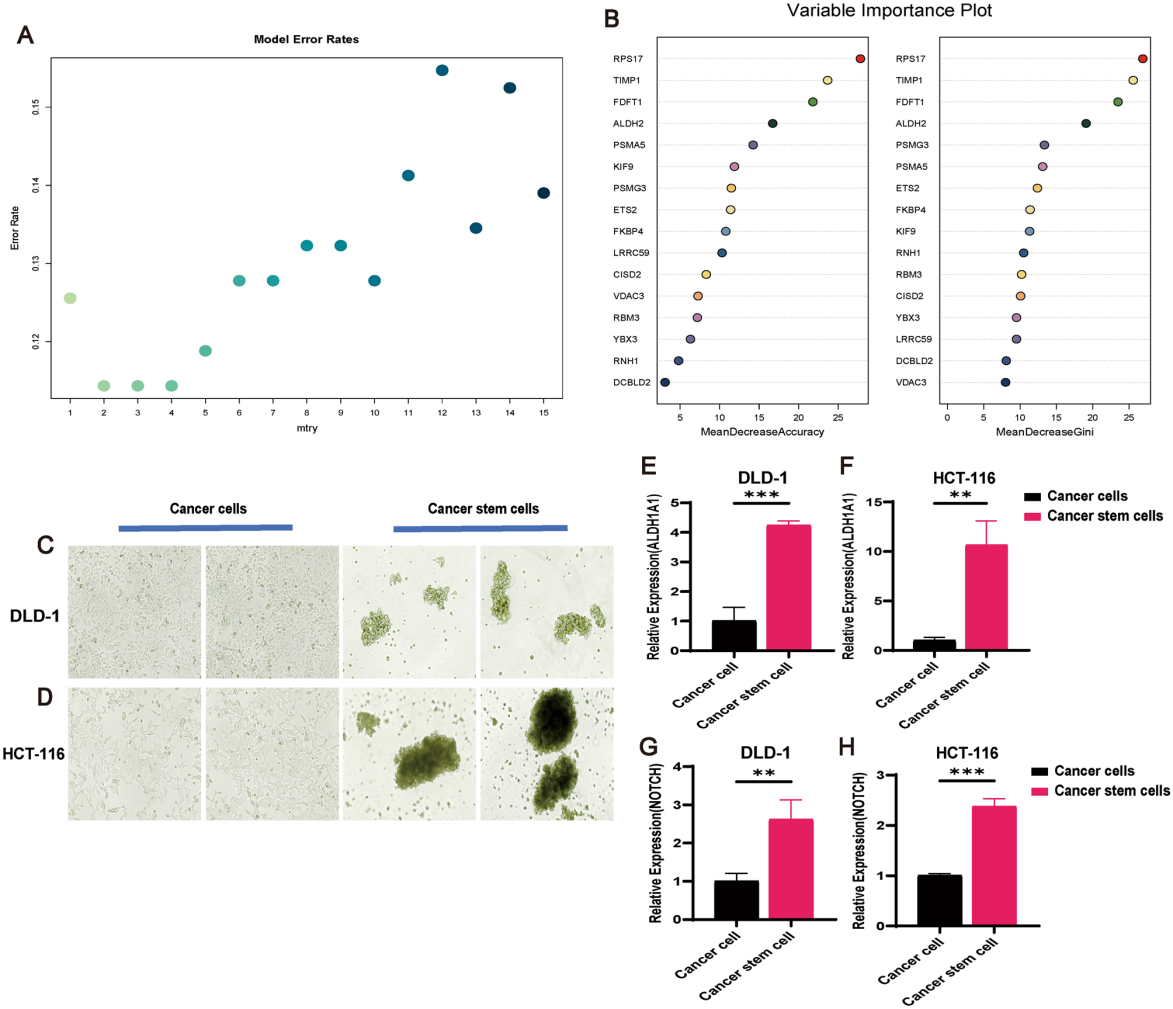
Validation of CRCSC-related genes by qRT-PCR. (**A**) Scatterplot identifies mtry = 4 as the optimal parameter for constructing the Random Forest model. (**B**) Random forest analysis highlights RPS17, TIMP1, ALDH2, FDFT1, PSMG3 and PSMA5 as key genes with stable contribution scores and significant model importance. (**C**,**D**) Morphological evidence of CSC enrichment in DLD-1 and HCT116 cells following a 7-day enrichment protocol. (**E**,**F**) qRT-PCR validation of increased ALDH1A1 expression in enriched CRCSCs (P < 0.05). (**G**,**H**) qRT-PCR validation of elevated NOTCH expression in enriched CRCSCs (P < 0.05).

To further explore the precise potential targets, we used the random forest method to analyze the expression matrix of the 16 genes in the risk model, and explored the CRCSCs-related genes with the top contributing values in the model to facilitate subsequent studies^77^. We chose mtry = 4 (Fig. 10A) to minimize the error rate in the forest plot, and the obtained random forest plot is shown in Fig. 10B. The results showed that we selected the top five MeanDecreaseAccuracy and MeanDecreaseGini, RPS17, TIMP1, FDFT1, ALDH2, and PSMG3/PSMA5, which were significant contributors and meaningful among the 16 risk-modeled genes (with a combined contribution rate of greater than 15%). To confirm the expression of the aforementioned six genes in CRCSCs, we initially isolated CRCSCs from DLD-1 and HCT-116 cells using serum-free sphere-forming culture methods (Fig. 10C,D). Subsequently, the expression of CRCSC markers ALDH1A1^78,79^ (Fig. 10E,F) and NOTCH^80,81^ (Fig. 10G,H) was assessed through qRT-PCR, validating that the isolated cells indeed exhibited characteristics of CRCSCs. The expression levels of RPS17, TIMP1, FDFT1, ALDH2, and PSMG3/PSMA5 in both CRCs and CRCSCs are illustrated in Supplementary Fig. 3. Interestingly, the expression levels of these six genes were notably elevated in CRCSCs compared to CRCs, suggesting a distinct association of these genes with CRCSCs. Multiple studies have documented the involvement of all 5 genes, except for RPS17, in tumor progression^82–86^. Conversely, RPS17 is uncommonly studied in cancer research, with limited reports focusing solely on its bioinformatics analysis in CRC, lacking relevant experimental investigations. Furthermore, there is scarce literature regarding its correlation with CRCSCs, and no significant variants of RPS17 have been identified. Hence, we selected RPS17 as a fundamental gene for our subsequent studies.

**Figure 11:**
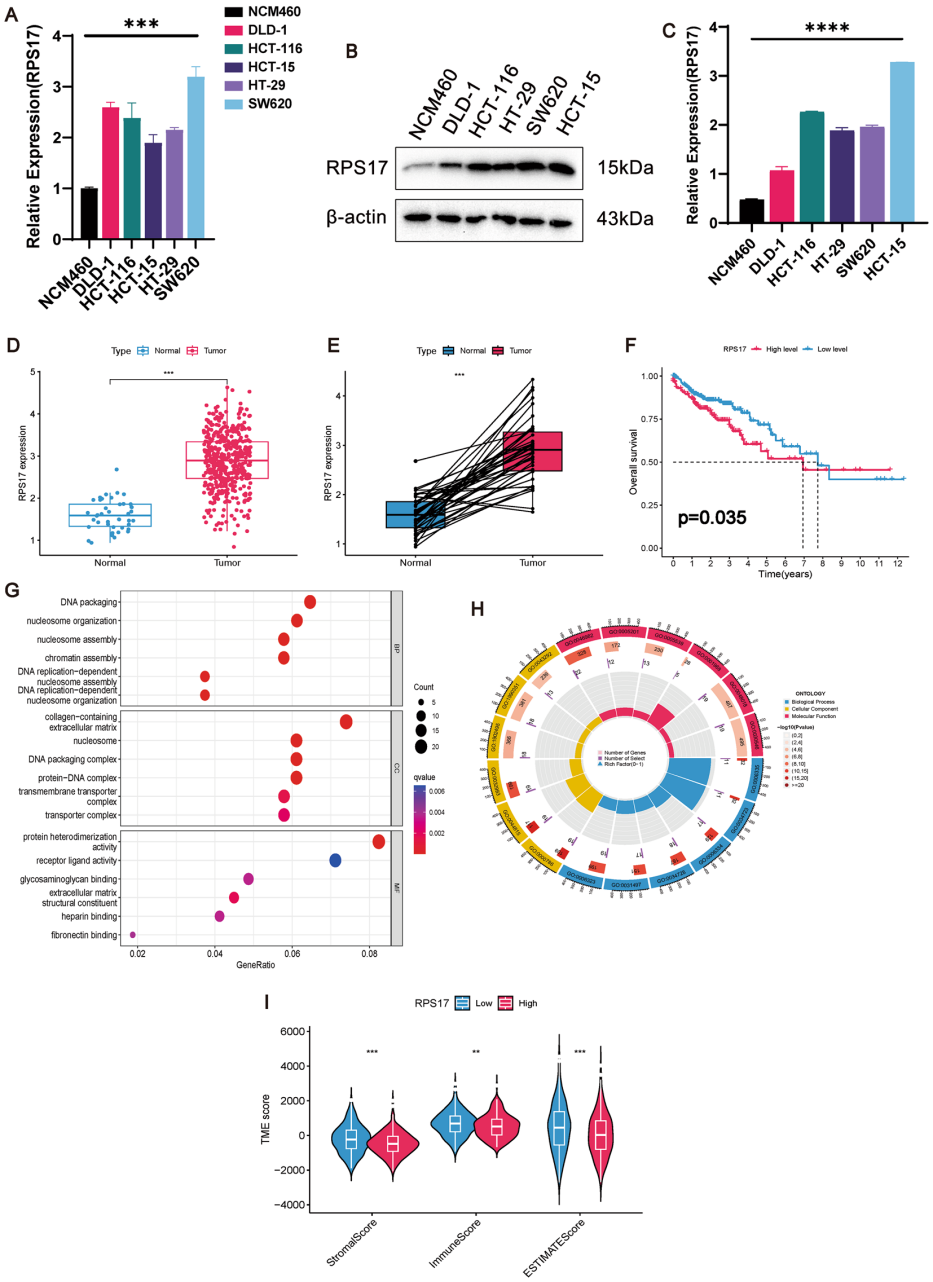
Experimental and mechanistic prediction of RPS17 expression in CRC. (**A**) qRT-PCR confirmed the high expression of RPS17 in CRC cell lines compared to normal colorectal epithelial cells, NCM460 for normal colorectal epithelial cells, and DLD-1, HCT116, HCT15, HT-29 and SW620 for CRC group cell lines, P < 0.05. (**B**) Western blot immunoblotting showed significantly elevated protein levels of RPS17 in CRC cells. (**C**) Western blot immunoblot statistical analysis plots with one-way ANOVA. (**D**) Differential analysis demonstrating that RPS17 is highly expressed in CRC. (**E**) Pairwise differential expression demonstrating that RPS17 is highly expressed in CRC. (**F**) Survival differential analysis demonstrating that the overall survival rate of patients with high expression of RPS17 is significantly lower than that of patients with low expression. (**G**) GO results show that RPS17 is enriched in DNA packaging during biological processes, and that cellular components are enriched in collagen-containing extracellular matrix and are active in the molecular function of protein heterodimerization activity, corresponding to the circle diagrams demonstrated in the (**G**). (**H**) Violin diagrams demonstrates that high expression of RPS17 leads to a decrease in both TME scores (including Stromal Score, Immune Score, ESTIMATE Score).

The results from qRT-PCR and Western blot analyses revealed a marked elevation in both mRNA (Fig. 11A) and protein (Fig. 11B,C, original blots are shown in Supplementary Fig. 4) expression levels of RPS17 in five CRC cells compared to normal colonic epithelial cells (NCM460). Meanwhile, the predicted results of TCGA dataset were also confirmed the high expression of RPS17 in CRC (Fig. 11D,E) with significant prognostic difference (P = 0.035) (Fig. 11F). The results of GO functional analysis (Fig. 11G,H) showed that its DNA packaging and the collagen- containing extracellular matrix, as well as demonstrating protein heterodimerization activity. In addition, the high expression of RPS17 was accompanied by a decrease in the TME score (Fig. 11I), which may indicate that the that RPS17 expression is associated with the heterogeneity of the TME.

## Discussion

The increasing incidence of CRC can be linked to diverse factors such as economic development, lifestyle choices, environmental changes, and genetic predisposition^87^. Within this context, CRCSCs represent a subset of cancer cells characterized by robust proliferation and limited differentiation abilities^88^. These cells propel tumor progression, support immune escape, and foster drug resistance through complex interactions and signaling pathways within the tumor microenvironment (TME), thereby influencing CRC's vulnerability to recurrence^89^. This phenomenon significantly heightens the risk of CRC recurrence, widespread metastasis, delayed detection, and unfavorable prognostic consequences^90^. Therefore, the exploration of new biomarkers associated with CRCSCs holds promise in providing essential clinical insights to enhance the diagnosis, prognosis, and management of CRC patients.

Based on the scRNA-seq GSE188711 public dataset, we profiled TMEs in CRC samples at the cellular level, identified 15 different cell subpopulations (Fig. 2A,B), and analyzed cellular communication, metabolic level, and differentiation trajectory for different cell subpopulations. In TME, cancer cells and CRCSCs shared similar marker genes, however, aberrant expression was more pronounced in CSC populations than in cancer cells (Fig. 2A–D), while we observed the highest number of interactions between CSCs and cancer cells (Fig. 3A,B), and the communication linkage between these two cell types involves EPHA/B. We also observed the highest number of interactions between CSCs and cancer cells (Fig. 3A,B), CDH/CDH1, DESMOSOME and OCLN signaling (Fig. 3C–F). It has been reported that EPHA/B is considered as a novel marker for CRCSCs and is associated with migration and invasion signaling between CSCs and cancer cells^91,92^. Additionally, CDH, DESMOSOME, and OCLN are known to play significant roles in maintaining cell adhesion and invasion^93–95^.

CRCSCs exhibit heightened metabolic activity, crucial for maintaining their self-renewal and environmental adaptability, ensuring constant energy and nutrient supply^96^. KEGG metabolic pathway analysis identified the top 30 active pathways within the TME, with CRCSCs significantly involved in 15 of these (Fig. 3G). These pathways include glycolysis, fatty acid oxidation, oxidative phosphorylation, and the TCA cycle, underscoring CRCSCs' substantial impact on TME metabolism. Interestingly, butyric acid and sulfur metabolisms were newly identified. Furthermore, a time series analysis, starting with CRCSCs, showed a greater differentiation toward cancer cells (Fig. 3H,I), suggesting a biologically significant exchange of signals, including cell adhesion pathways^97^, between CRCSCs and cancer cells, essential for regulating CRC growth and invasion.

Following univariate Cox regression, we conducted subsequent analyses involving prognostic-related consistency assessment and Lasso-Cox regression to establish a risk model encompassing 16 genes associated with CRC stem cells (CISD2, RNH1, DCBLD2, VDAC3, ALDH2, YBX3, FDFT1, RBM3, FKBP4, PSMG3, LRRC59, KIF9, TIMP1, ETS2, PSMA5, and RPS17) (Fig. 5A,B). This risk model was then utilized to calculate the median for predicting and diagnosing the prognosis of CRC patients. Our results highlight a significantly lower survival rate in the high-risk group compared to the low-risk group (Fig. 6I,J). Further assessments, including Principal Component Analysis (PCA) (Fig. 6A,B), ROC curve analysis (TCGA: Fig. 6C,D; GEO: Fig. 6E,F), and validation via nomogram and calibration curve (Fig. 6G,H), have collectively provided strong validation. Additionally, our confirmation of the risk prognostic model's potential as an independent prognostic factor (Fig. 6I,J) underscores the successful development of a prognostic risk model rooted in genes relevant to CRC stem cells, serving as a theoretical guide for CRC patients in a clinical setting.

Notably, the GO functional analysis confirmed that our prognostic risk model is significantly associated with the WNT signaling pathway, which is known to advance CRC progression, metastasis, and recurrence by modulating CRCSCs self-renewal^98,99^. HALLMARK analyses further indicated that the model is enriched in epithelial–mesenchymal transition (EMT) and E2F pathways. EMT, a key factor in maintaining stem-like properties in cancer cells, is crucial for metastasis and invasion in CRC and other cancers^100^. This suggests that patients identified as high-risk by the model may present more severe CRC symptoms. E2F, which promotes CRCSCs proliferation and self-renewal^101^, has been shown to reduce cancer cell invasiveness when less active, contributing to slower cancer progression^102,103^. This implies that low-risk patients, as determined by the model, may have milder CRC symptoms. These findings validate the model's accuracy in diagnosing functional pathways related to CRCSCs and its potential for differentiating between high- and low-risk CRC patients.

Although immune recognition and suppression of cancer cells show promise as strategies for advanced cancers^104^, TME exhibits immunosuppressive properties, where interactions between CSCs and immune cells play a pivotal role in this context^105^. Research suggests that CSCs release mediators that direct monocytes to tumor niches, facilitating the differentiation of macrophages into tumor-associated subsets^106^. Moreover, CSCs can recruit regulatory T cells using chemokines such as CCL1, CCL2, and CCL5^107^, thus disturbing immune homeostasis and promoting self-tolerance and inflammation. The association between the removal of dysfunctional CD8+ T cells by CSCs and heightened stemness in breast cancer cells warrants further investigation^108^. Our analysis of cell communication revealed a substantial interaction between CRCSCs and various immune cells, such as macrophages, B cells, and CD8+ T cells (Fig. 3A,B), showcasing the potential interplay between CSCs and distinct immune cell populations. Detailed analysis of CRCSC-related risk model and immune responses (Fig. 8) demonstrated that patients at higher risk exhibit lower TIDE scores, suggesting reduced potential for immune evasion and potentially improved outcomes with immunotherapy (Fig. 8A). Single-sample gene set enrichment analysis (ssGSEA) revealed enriched type I and II IFN responses, cytolytic activity, and HLA expression in high-risk patients (Fig. 8B). These findings indicate immune response modifications associated with immune evasion mechanisms and CRC progression. Analysis of immune cell infiltration (Fig. 8C) revealed differences in T-cell CD4 memory quiescence, NK cell activation, macrophage M2 polarization, and neutrophil levels among distinct risk groups. Significantly, a decrease in the proportion of resting CD4+ memory T cells within the high-risk group could potentially increase immune evasion capabilities and reduce treatment efficacy^109^. Neutrophils and macrophages that are overactivated have the potential to release factors such as GM-CSF, TNF, and ILs, which promote the growth and dissemination of CRC cells, consequently contributing to recurrence and metastasis risks in high-risk patients^110,111^. Activated NK cells are well-known for their ability to inhibit the development and progression of CRC by leveraging their tumoricidal functions. Immunotherapies directed at NK cells significantly contribute to improving the prognostic evaluation for CRC patients^112,113^. Despite the effectiveness of NK cells in eliminating CSCs, the potential for CSCs to evade immune responses remains a concern in the context of NK cell-based therapies^114^. Prognostic models utilizing the CRCSC-associated risk model can efficiently assess the immune status of patients, thereby guiding the development of personalized immunotherapy strategies.

In the domain of CRC clinical management, chemotherapy stands as the cornerstone, where its therapeutic effectiveness relies significantly on cancer cell susceptibility to chemotherapeutic agents^115^. This highlights the necessity of investigating drug sensitivity within prognostic risk models. Our study reveals that high-risk patients exhibit reduced IC50 values for cisplatin, XAV939, Elesciomol, and (5Z)-7-oxozeaenol compared to low-risk patients (Fig. 9A), indicating diminished responsiveness to these medications. Notably, cisplatin, a commonly utilized anticancer drug, faces challenges linked to resistance and toxicity^116^. XAV939, targeting the Wnt/β-catenin pathway through increased β-catenin degradation, presents a promising therapeutic approach^117^. The preclinical promise of Elesciomol and (5Z)-7-oxozeaenol in CRC, while still uncharacterized, justifies deeper exploration. Furthermore, the direct association between the IC50 values of AC220 and Genentech Cpd 10 with risk model scores implies the potential requirement of elevated dosages for optimal therapeutic outcomes. These discoveries establish a conceptual framework and indicate novel avenues for the preclinical and clinical utilization of chemotherapeutic agents.

Through meticulous analysis, we have developed a robust prognostic model associated with CRCSCs. This model efficiently stratifies patients, exhibiting potential to significantly improve diagnostic precision in clinical settings. Moreover, it surpasses other variables in predictive accuracy for outcomes, addressing critical clinical challenges like low early detection rates, delayed symptom presentation, and suboptimal CRC prognoses. Interestingly, our risk model identifies notable variations in immune evasion, immune response, and treatment efficacy in immunotherapy, suggesting its value in guiding immunotherapeutic approaches. In sum, our prognostic model demonstrates promising machine learning forecasts and the potential to emerge as a novel CRC biomarker pending further preclinical validation. This progress not only enriches our understanding of CRCSCs but also paves the way for personalized and effective therapeutic strategies.

Investigating the intricate regulatory mechanisms driving CRC development, we utilized a random forest approach to pinpoint critical genes significantly impacting the CRC risk model (Fig. 10A,B). We identified the top six genes linked to CRC stem cells (RPS17, TIMP1, ALDH2, FDFT1, and PSMG3/PSMA5) with the highest contribution values to the model. Through qRT-PCR verification, we confirmed the association of these genes with CRCSCs and observed distinct expression patterns; specifically, FDFT1 exhibited lower expression in DLD-1 CSCs but higher expression in HCT116 CSCs, while PSMA5 displayed contrary expression profiles between the two cell lines. These variations may arise from microenvironmental adaptations within CSCs or genetic and epigenetic regulations across different cell lines^118^. For our subsequent investigation, we selected RPS17 due to its novel and substantial contribution value, an aspect unexplored in previous studies. Initially, we examined the potential influence of alternative splicing on CRCSCs' heterogeneity and prognostic modeling. Our search on NCBI indicated that RPS17 possesses exclusive coding transcripts. Remarkably, there exists no literature investigating alternative splicing events directly linked to RPS17. Notably, RPS17 exhibited elevated expression at both molecular and protein levels in comparison to CRC (Fig. 11A–C), aligning with our expectations, and heightened expression correlated with diminished overall survival rates (Fig. 11D–F). Further functional analysis (Fig. 11G–I) and TME scores hinted at RPS17’s potential impact on CRC progression.

In summary, we have developed a prognostic signature comprising 16 novel CRCSCs-related genes through the integration of scRNA-seq and Bulk RNA-seq analysis. The validation of this signature demonstrated its effectiveness in predicting the prognosis of patients with CRC, positioning it as an independent prognostic factor for CRC patients. Furthermore, our findings shed light on the potential involvement of RPS17 in the regulation of CRCSCs. These results not only establish a new theoretical foundation for refining CRC treatment strategies but also pave the way for novel research avenues aimed at unraveling the molecular underpinnings of CRC onset and progression.

## Methods

### Data collection and pre-processing

The scRNA-seq dataset GSE188711^119^ is from the public database GEO (Gene Expression Omnibus) and includes 6 CRC tissues (3 left colorectal CRC and 3 right CRC tissues).The transcriptome dataset (TSV format) and clinical information data (Clinical data from 446 patients were selected after screening, XML format) includes from the TCGA (The Cancer Genome Atlas) dataset (524 samples, including 42 normal and 482 tumor samples), The dataset was organized via Perl v5.30.0. GSE39582^120^ (containing 585 samples including 19 normal and 566 tumors samples, Clinical data from 579 patients were selected after screening, XML format), GSE33113^121^ (containing 96 samples including 6 normal and 90 tumour samples)). We utilized Perl scripts to preprocess the raw GEO data into RNA-seq matrices and extract relevant clinical data for an independent validation set in future prognostic modeling. To mitigate data errors from the combined analysis of TCGA and GEO datasets, we harmonized both datasets using the “limma” and “SVA” R packages. The GEO dataset underwent log transformation (log2(X + 1)). To ensure gene consistency between TCGA and GEO, we identified shared genes, integrated them into a new data frame, addressed batch effects, and applied the “Combat” function to minimize batch-related biases in the analysis results. After the clinical samples were name-consistent, the Train and Test datasets were merged and looped for each clinical variable, the frequencies and proportions of each variable were counted for the different types of data (training and test groups), and Fisher's exact test was performed to assess the association between them.

### Single-cell data analysis

Single-cell RNA sequence data from 6 samples were analyzed while studying the GSE188711 (including 19,872 CRC cells) dataset for CRC. The “Seurat” R package^122^ was utilized to construct S4 objects, and then the “Harmony” R package^123^ was used to integrate data from multiple samples. To ensure data quality, the following screening conditions were set: (1) Each gene has expression in at least 3 cells. (2) The genetic count per cell ranges from 300 to 7000. (3) No more than 10% of mitochondrial genes are expressed. (4) The proportion of human blood-derived genes expressed was at least 3%. (5) Cell cycle related genes are excluded. (6) Total RNA counts are less than 100,000. The QC visualization is shown in Supplementary Fig. 5. For enhanced analysis accuracy, we initially normalized the raw expression values in the scRNA-seq data using the NormalizeData function to mitigate sequencing depth and technical discrepancies. Subsequently, the FindVariableFeatures function detected genes displaying significant biological variability across cells using the “vst” parameter, followed by normalization of gene expression within cells via the ScaleData function. Dimensionality reduction was performed through the RunPCA function, while cell clustering, with a resolution of 0.5, was achieved using FindNeighbors and FindClusters with dims = 1:20 settings. Visualization of cell distribution was conducted using the RUNTSNE function.

### Cell communication analysis

To gain insight into the communication and interactions between 13 cell types, including CSCs, we used the “CellChat” R package^124^ to analyze the intercellular communication networks. This package reveals how different cell populations interact with each other through secreted factors and receptors, with a special focus on the communication network of CSCs. After analysis with CellChat, we used the “NMF” R package to perform unsupervised clustering of those communication networks to identify the communication patterns into several different modules. To determine the optimal number of modules, we called the selectK function, which evaluates the robustness of different numbers of patterns by computing two metrics, Cophenetic and Silhouette. These metrics are computed based on hierarchical clustering of the consensus matrix and help us identify the most reasonable number of modules to choose at the sudden drop in Cophenetic and Silhouette scores. By drawing heatmaps and flow diagrams, we visualize the incoming and outgoing communication patterns between cells.

### Cell trajectory analysis

To simulate and understand the differentiation trajectories between cells, we used the “monocle” R package^125^ to perform the proposed time-series analysis and constructed the “umap” scatter plot with CSCs as the reference starting point, which outlines the potential differentiation pathways between different cells.

### Cellular metabolic enrichment analysis

To investigate the metabolic activities of 13 different cell types in TME, we used the “scMetabolism” R package^27^, based on the human species “KEGG” database^126^ via “AUcell” as a metabolic pathway activity scoring method, to explore potential metabolic enrichment pathways between different cells and to gain a preliminary understanding of metabolism in the complex TME.

### Constructing consensus clustering associated with CSC-related genes

In a prognostic study of CRC patients, we used the TCGA-COAD database to identify marker genes for CSCs associated with prognosis by the univariate Cox regression analysis, with P < 0.05 as the significance criterion. Subtype clustering was then performed using the “ConsensusClusterPlus” R package with the parameters reps = 50, pItem = 0.8, pFeature = 1, and the distance was adopted from “Euclidean”. The optimal number of clusters k is determined by the CDF curve and the consensus matrix. Finally, the “Survival” package was used to evaluate the effect of different CSCs typologies on survival.

### Prognostic risk model construction and validation

To better investigate the effect of CSC-related genes on the prognosis of CRC patients, we used the “glmnet” R package in R to perform LASSO (least absolute shrinkage and selection operator) regression analysis. The TCGA dataset was used as the training group, and the GSE39582 dataset was used as the test group. In the training group, we utilized univariate Cox regression analysis to screen for prognostically relevant CSC gene differences (P < 0.05). Then, we used the LASSO regression analysis formula to calculate the LASSO coefficients of CSC genes and the expression levels of prognostic genes (Number of randomized cycles of the model: 1000). The formula is as follows: (Exp: the expression level of prognostic genes; Coe: the lasso coefficient.)

$${\text{Risk Score}}={\sum }_{{\text{K}}=1}^{{\text{N}}}\left({\text{Expk}}*{\text{Coek}}\right)$$

Based on the calculated median risk score, we divided the training set into high and low risk groups and the test set served as validation. The “Survival” package was used to explore the survival differences between high and low risk groups and to investigate the independent prognostic ability of the risk prognostic model. “timeROC” package was used to construct prognostically relevant ROC curves. The “Regplot” package plotted Nomograms and 1-, 3-, and 5-year prognostic prediction outcome correction curves. The “Regplot” package is used to plot Nomogram and 1-, 3-, and 5-year prognostic calibration curves.

### Functional enrichment analysis

We screened differentially expressed genes (DEGs) from the sample features using |log2FC| ≥ 1 and P < 0.05 as criteria. To reduce the possible errors between the scRNA-seq dataset and the traditional transcriptome dataset, after initially screening these differentially expressed genes, we plotted the volcano map of the GSE33113 dataset using the “EnhancedVolcano” package to visualize the expression patterns of these DEGs. After that, we enriched and analyzed the DEGs using a series of R packages such as “org.Hs.eg.db”, “clusterProfiler”, and “enrichplot”. We focused on the differential enrichment in GO (Gene Ontology), KEGG (Kyoto Encyclopedia of Genes and Genomes) database^126^ and Hallmark (Hallmark Genomes) and used R = 1, P < 0.05 as the screening threshold.

### Immunological correlation analysis

To explore the function of the CSC-related prognostic model in terms of immunity, we performed a variety of immune analyses. Firstly, we performed an analysis of immune-related functions and assessed the immune escape ability between the high-risk and low-risk groups using TIDE (Tumor Immune Dysfunction and Exclusion) immune escape score analysis^127^, the TIDE analysis is realized by the “limma” R package. In addition, we also performed enrichment analysis of immune-related functions between the high- and low-risk groups by ssGSEA (Single Sample Gene Set Enrichment Analysis) analysis of 13 immune-related pathways. Immune-related functions were assessed using GSVA via the gsva function, and the scores underwent normalization to generate corrected data after excluding normal samples. These scores were then integrated with the risk model for variance analysis and visualization using the pheatmap function. Additionally, immune infiltration analysis was conducted utilizing the “CIBERSORT” R package in conjunction with model expression and risk modeling, following the removal of normal samples. The analysis utilized the “KEGG” database with a significance threshold of P < 0.001. The Wilcoxon signature test was also conducted.

### Drug sensitivity analysis

To predict changes in IC50 (biochemical half-maximal inhibitory concentration) in risk-based prognostic modeling for chemotherapeutic agent sensitivity, drug sensitivity prediction was conducted using the “pRRophetic” R package. This involved iterating through all drugs, integrating the risk model and drug sensitivity results sequentially, followed by various statistical analyses (Wilcoxon test and correlation analysis). Subsequently, drugs exhibiting a significant correlation (P < 0.001) between the difference and the correlation were filtered out, considering only drugs meeting this criterion for further visualization.

### Cell culture and CSCs enrichment formation assay

In this study, NCM460, DLD-1, HCT-116, SW620, HT-29 and HCT-15, were originated from the laboratory of the Chinese University of Hong Kong. All cell lines were cultured in Duchenne’s modified Eagle's medium (Procell) containing 10% foetal bovine serum (FBS, from Guangzhou Haoguo Biotechnology Co., Ltd.), 1% penicillin–streptomycin (Beyotime), and were maintained at 37.0 °C and 5.0% CO_2_. cells were seeded at a density of 1 × 10^7^ in 10 cm^2^ cell culture dishes for 7 days and cultured through CSCs-specific medium, which was formulated^128^. After 7 days, suspended CSCs (spherical, non-adherent mass) were collected.

### qRT-PCR

Cellular RNA was extracted using AG RNAex Pro Reagent (Agbio, Changsha, China), and cDNA was obtained after reverse transcription with Evo M-MLV RT Premix (Agbio, Changsha, China), and real-time quantitative polymerase chain reaction (qRT-PCR) was carried out by SYBR Green Premix Taq HS qPCR kit (Agbio, Changsha, China). The qRT-PCR primers used in this study are shown in Table 3.

**Table 3:** The qRT-PCR primer sequences used in this study.

Gene	Primer
RPS17	F: 5′-GAGAGGCCCAGTAAGAGAGGTA-3′
	R: 5′-GACCTGAAGGTTGGACAGACAC-3′
TIMP1	F: 5′-CTTCTGCAATTCCGACCTCGT-3′
	R: 5′-ACGCTGGTATAAGGTGGTCTG-3′
ALDH2	F: 5′-ATGGCAAGCCCTATGTCATCT-3′
	R: 5′-CCGTGGTACTTATCAGCCCA-3′
FDFT1	F: 5′-GGACTCGACAGACTCTAAGGC-3′
	R: 5′-CAATAAGTCGCCCACGTGTC-3′
PSMG3	F: 5′-GAAGACACGCCGTTGGTGATA-3′
	R: 5′-GAAGGACTTTTGTGGTGAGCA-3′
PSMA5	F: 5′-TGCCATGAGTGGGCTAATTG-3′
	R: 5′-GGCACCTGGATCTGCATCTT-3′
ALDH1A1	F: 5′-ACTTACCTGTCCTACTCACCGA-3′
	R: 5′-CTGTCTTGCGGCCTTCACT-3′
NOTCH	F: 5′-ACTGCGAGGTCAACACAGAC-3′
	R: 5′-GTCCACATCGTACTGGCACA-3′

### Western blot

Proteins were separated by adding protease inhibitor (Kangway, CW2200S) in ice-cold RIPA buffer (Solarbio, R0020) and protein concentration was determined by bicinchoninic acid assay (BCA, Beyotime, P0012). PAGE Gel Rapid Preparation Kit (15%) Polyacrylamide Gelatins were prepared (EpiZyme, PG114), proteins were electrophoresed, transferred to a PVDF membrane (polyvinylidene difluoride membrane) and detected with primary and secondary antibodies. Primary antibodies used: RPS17 (Sinobiological, 202778-T46), β-actin (Servicebio, A2317). Protein bands detected by the antibodies were visualized by enhanced chemiluminescence (Beyotime, P0018FM-2) and evaluated using Image J. The antibodies were used to detect the protein bands.

### Statistical analysis

This study was mainly analyzed using R studio (version 4.2.2) and GraphPad Prism 9 was used as a statistical analysis tool for qRT-PCR, P < 0.05 was considered statistically significant (*P < 0.05, **P < 0.01, ***P < 0.001).

### Supplementary Information

Supplementary Figures.

## Data Availability

In this study, datasets based on TCGA database (GDC (cancer.gov)), GEO database (Home—GEO—NCBI (nih.gov)), GSEA database (GSEA (gsea-msigdb.org)), TIDE database (http://tide.dfci.harvard.edu/) and Figdraw were used. In the GEO database, the dataset accession numbers used were GSE188711 (GEO Accession viewer (nih.gov)), GSE39582 (GEO Accession viewer (nih.gov)) and GSE33113 (GEO Accession viewer (nih.gov)).
