# Supplementary material for: Integration of single-cell sequencing and bulk RNA-seq to identify and develop a prognostic signature related to colorectal cancer stem cells
Source: Sci Rep. 2024 May 28;14:12270. doi: 10.1038/s41598-024-62913-3 (PMC11133358; doi:10.1038/s41598-024-62913-3)
Supplement: Supplementary file 1 — Supplementary Figures. [file 41598_2024_62913_MOESM1_ESM.pdf]

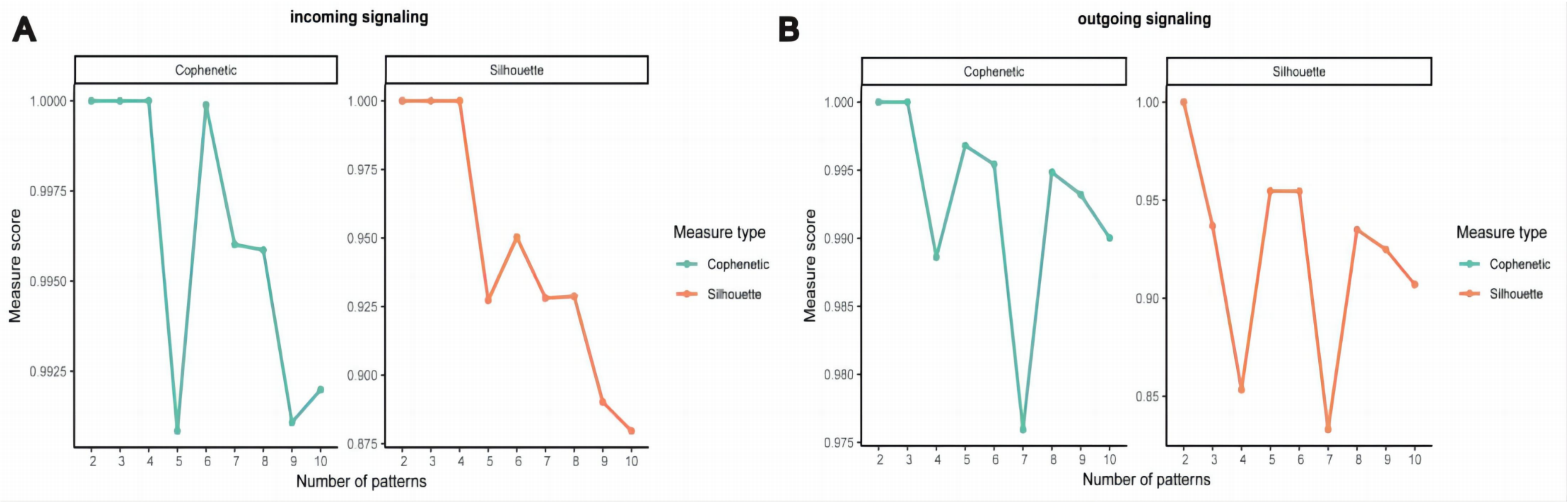

Supplementary Figure 1: The "NMF" package was used to construct Cophenetic and Silhouette curves to predict the number of cellular communication pattern. (A) Prediction curves for the number of patterns for Incoming signaling. (B) Prediction curves for the number of patterns for Outcoming signaling.

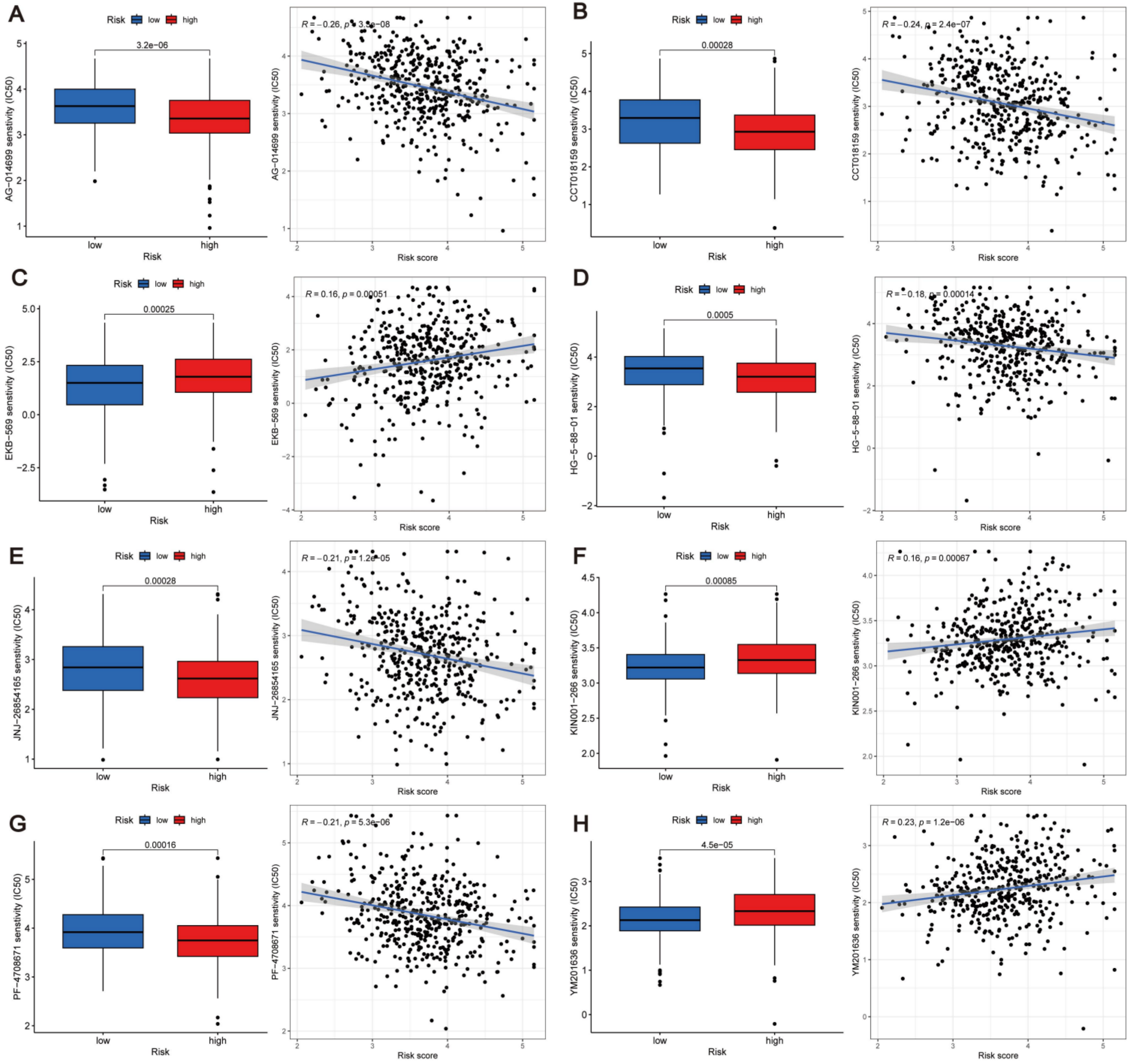

Supplementary Figure 2: Remaining chemotherapeutic drug sensitivity and correlation prediction results. (A) AG-014699; (B) CCT018159; (C) EKB-569; (D) HG-5-88-01; (E) JNJ-26854165; (F) KIN001-266; (G) PF-4708671; (H) YM201636.

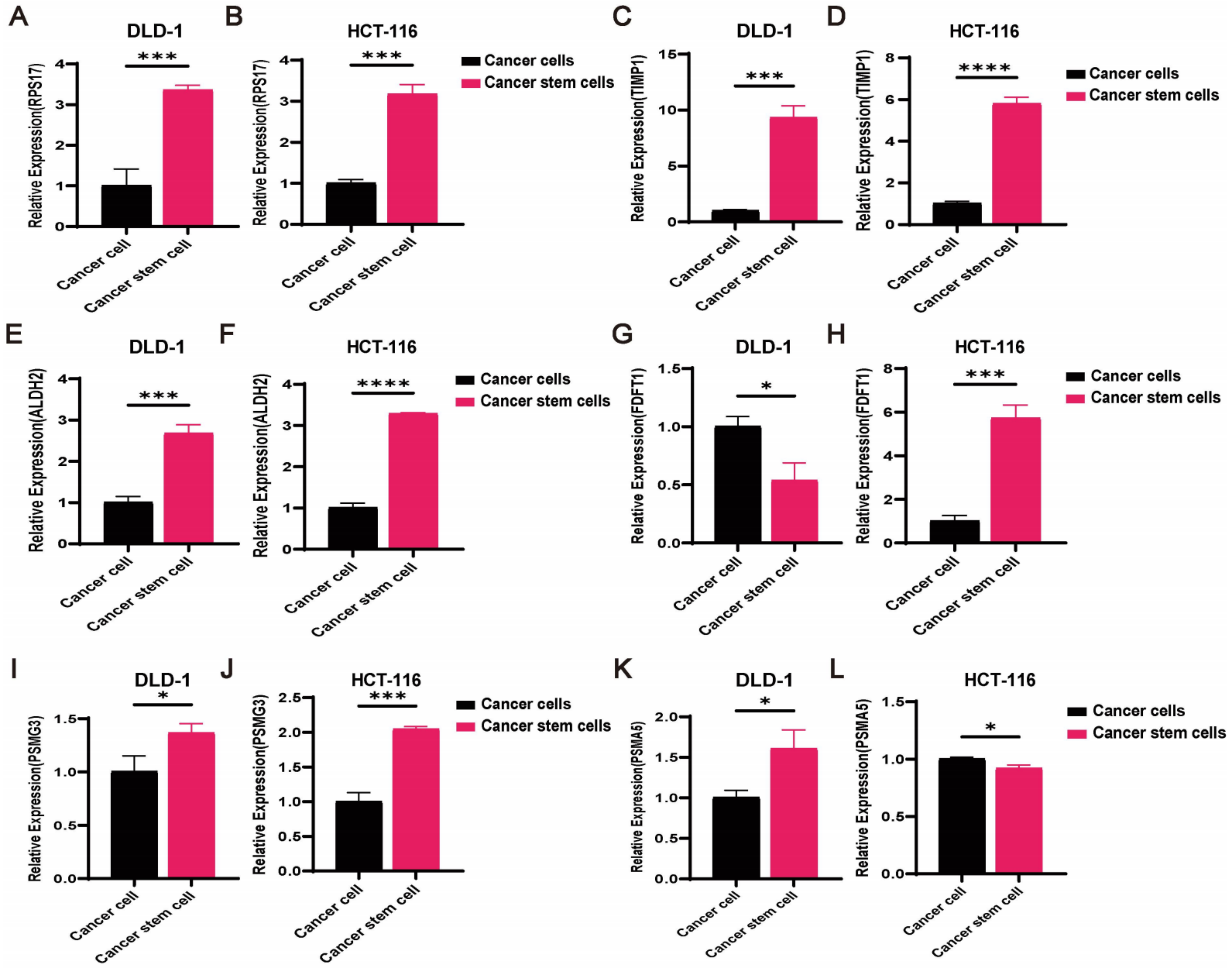

Supplementary Figure 3: qRT-PCR validation of enhanced expression ($P<0.05$) of key genes associated with CRCSCs in enriched CRCSCs,(A,B) RPS17. (C,D) TIMP1.(E,F) ALDH2.(G,H) FDFT1.(I,J) PSMG3. (K,L) PSMA5.

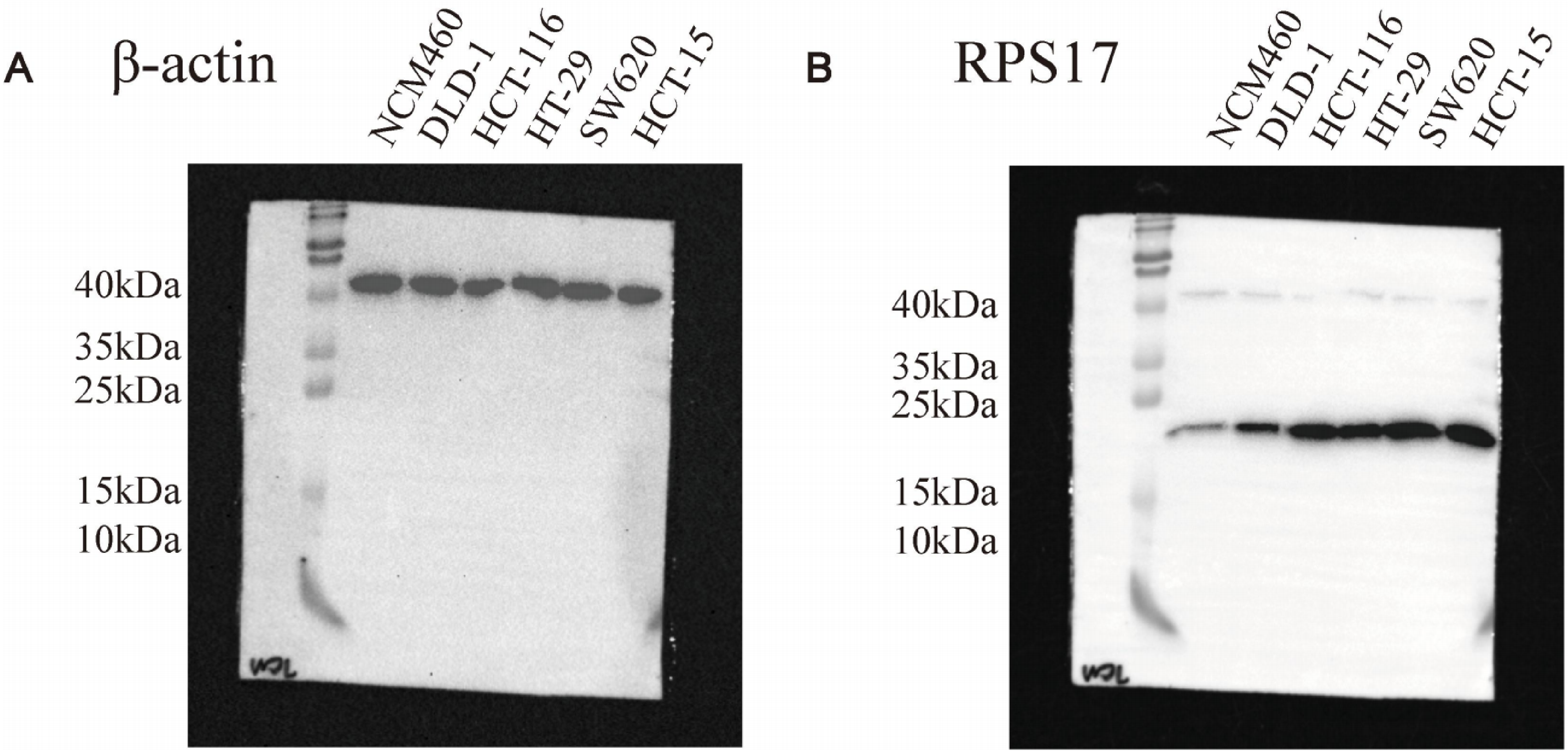

Supplementary Figure 4: Western blots original images. (A) β-actin.(B) RPS17.

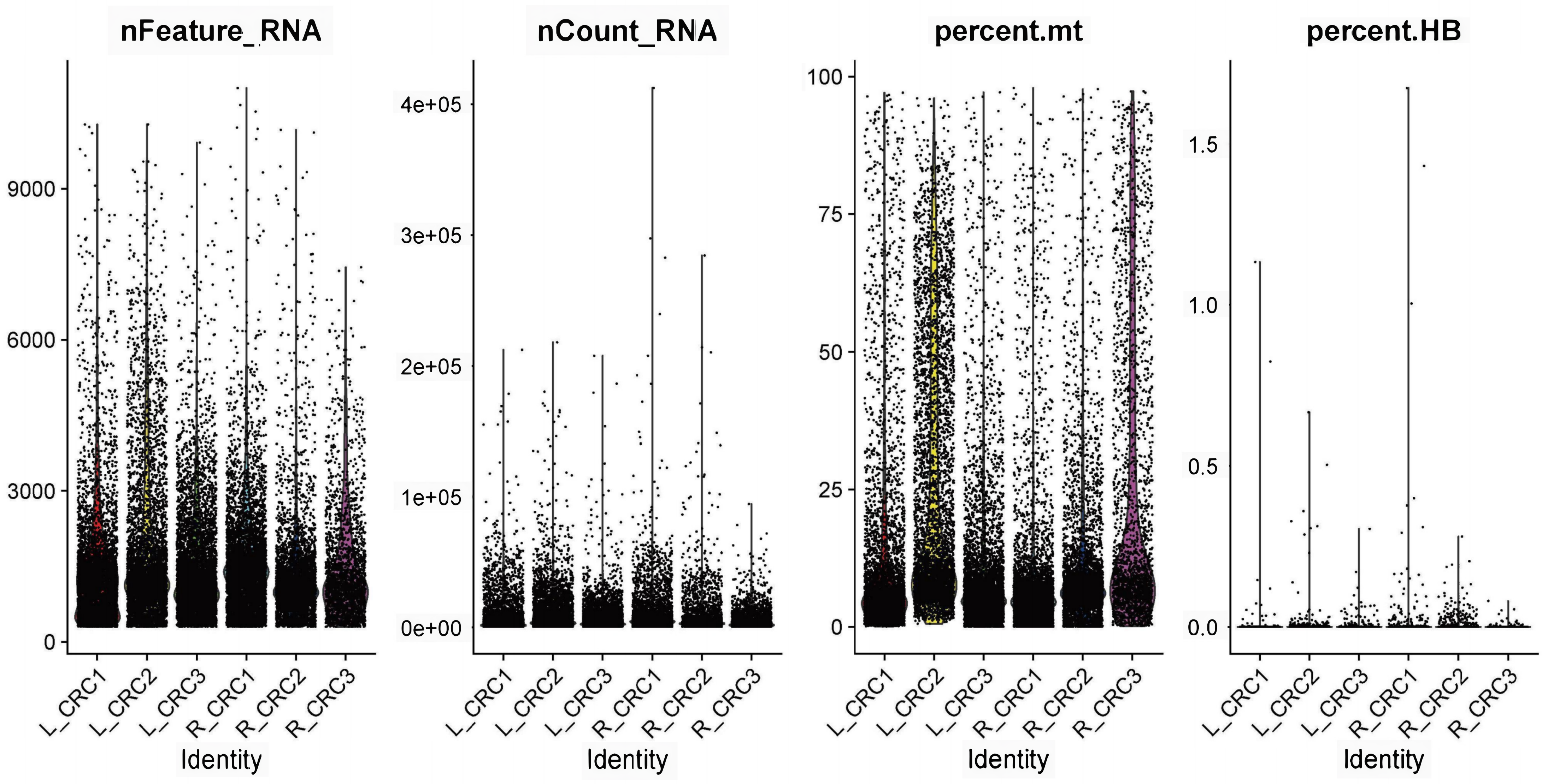

Supplementary Figure 5: Visualization of quality control for single-cell datasets.
